# Blockchain-enabled strategic optimization: A decision analytics framework for multinational enterprises’ e-commerce entry model

**DOI:** 10.1371/journal.pone.0335655

**Published:** 2026-05-08

**Authors:** Liurui Deng, Xiaoying Hu, Ying Xiao

**Affiliations:** 1 College of Business, Hunan Normal University, Changsha, Hunan, China; 2 School of Economics and Trade, Hunan Vocational College of Commerce, Changsha, Hunan, China; University of Economics Ho Chi Minh City, VIET NAM

## Abstract

With e-commerce booming, MNEs (Multinational Enterprises) often tap into online markets through E-retailers in multinational e-commerce platforms. Therefore, which e-commerce entry model MNEs choose can be critical, especially when the MNE owning a retail division in the same market. Notably, the MNE can address the plague of low-quality images of e-retailers by introducing blockchain technology, but the effectiveness of blockchain technology for quality verification also varies across different e-commerce entry models. In this paper, we develop a two-tier supply chain model with a manufacturing division in a high-tax area and various retailers in a low-tax area. The retailers comprise the manufacturing division of the MNE and e-retailers. The e-retailers include self-operated e-commerce, FBP merchants, i.e., merchants handling sales while the platform manages product delivery and after-sales services, and SOP merchants, i.e., merchants separately manage operations, shipping, and sales. We find that the larger the tax disparity, the lower the overall profits of MNEs, irrespective of the implementation of blockchain technology. Furthermore, the overall profit of the MNE is more significant when adopting a singular e-commerce entry model without the introduction of blockchain technology; conversely, the total profit of the MNE is more significant when adopting a composite e-commerce entry model with the introduction of blockchain technology. Interestingly, when blockchain technology is implemented, the MNE is more lucrative under the e-commerce entry model with a medium initial market potential if the service gap between the e-retailer and the manufacturing division is modest.

## Introduction

With the progression of global economic integration, cross-border e-commerce has emerged as a crucial component of international trade, and the global e-commerce sector is seeing substantial expansion. Worldpay predicts that by 2025, the worldwide e-commerce transaction volume will attain $8.3 trillion, with the Asia-Pacific area accounting for $4.33 trillion. The swift advancement of cross-border e-commerce has established it as a predominant trend for global brand suppliers to engage with e-commerce platforms to expand online markets and enhance product sales [1]. Consequently, e-retailers have diversified their purchasing strategies for products from global brands, reselling them on platforms such as eBay, Paytm Mall, Tmall, and Jingdong Global.

E-retailers are progressively broadening their competitive dynamics and market entry strategies. Amazon offers third-party retailers a presence model through the Platform Open Plan (POP), enabling them to establish their businesses on the Amazon platform. In the e-commerce entry model (POP), merchants may opt for the FBP (Fulfilled by POP) model, wherein their products are stored in the platform’s warehouse, with merchants handling sales while the platform manages product delivery and after-sales services. Furthermore, merchants can select the SOP (Sale on POP) model, which entails the e-commerce platform serving solely as a sales venue while merchants separately manage operations, shipping, and sales [2]. Secondly, an increasing number of e-commerce platforms have introduced their products to compete with the Self-operated e-commerce (E) model. An illustrative instance is Amazon, which, as a retailer, directly sells items and manages buying, inventory, order processing, shipping, and after-sales care, with products often marked “Ships from and sold by Amazon.com.” The many entry models provide multinational enterprises (MNEs) with more outstanding options while simultaneously augmenting the complexity of decision-making.

Although the development of the Internet and information technology, as well as the popularization of smartphones, have improved the transparency and fairness of shopping and provided consumers with a more convenient and efficient way of shopping [3], global branded products sold on e-commerce platforms are often plagued by the inability to gain the trust of consumers and the low quality of their image [4]. These challenges primarily originate from the lack of market regulation, product traceability information that cannot be assured, product quality and authenticity being questioned, and lousy product experience [4–6]. As for the emergence of blockchain technology, it presents fresh ideas for overcoming these difficulties. Blockchain technology offers the features of non-tampering and traceability, which may effectively guarantee the validity and integrity of product information [7]. From product traceability, blockchain technology can record every link of the product from production to sales. Consumers can scan the QR code on the product or visit a specific webpage to view detailed information about the product, including the source of raw materials, production process, transportation path, etc. [8], which improves the service experience of consumers for online products; from the aspect of smart contracts, blockchain technology can automatically process the processes such as orders, payments, and after-sales services, using verified and tamper-proof digital identity information on the blockchain [9], reducing human error and fraud, and increasing consumer trust in and utility of online products. Blockchain technology enables a safer and more transparent transaction environment for e-commerce platforms and ensures a high-quality image for e-retailers, improving their sales.

The adoption of blockchain technology provides e-commerce platforms with a more secure and transparent transaction environment, ensuring the high-quality image of online retailers. This can be specifically examined through its impact on consumers’ valuation of e-commerce products and their perception of service optimization by online retailers. Alba et al. (2025) [10]found that simulation results of willingness-to-pay distributions indicate consumers exhibit a median marginal willingness-to-pay of approximately $1.45 for traceability information compared to no traceability information. If this information is blockchain-verified, consumers are willing to pay an additional $0.33–0.38. Duan and Zhu (2025) [11] discovered that decentralized blockchain transparency significantly alters consumer interaction patterns, making them more inclined to engage with and select responsible actors who provide transparent information. In other words, consumers perceive blockchain traceability not only as proof of product quality but also as an additional service guarantee offered by retailers. This indicates that blockchain technology’s impact on consumer utility possesses dual attributes as both a “quality verification” mechanism and a “service-enabling” tool.

The predominant e-commerce entry models in the industry include “Self-operated e-commerce merchants only (E only),” “FBP merchants only (FBP only),” “Self-operated e-commerce merchants +FBP merchants (E+FBP),” and “Self-operated e-commerce merchants +FBP merchants +SOP merchants (E+FBP+SOP).” When the MNE selects single-type e-commerce entry models such as “E only” or “FBP only,” the introduction of blockchain technology has a limited impact on enhancing the image of e-retailers. In this case, the e-commerce platform already possesses high social recognition and trust, and its original quality endorsement has conveyed sufficient signals, making blockchain a redundant signal. At this point, the e-commerce market exhibits substantial initial market potential for which blockchain technology cannot significantly contribute. Rapezzi, M et al. (2025) [10] found in their experiments that since FBP merchants already benefited from the platform’s unified quality inspection and insurance, introducing blockchain only increased consumers’ willingness to pay a premium by 1.6%, far below the industry average of 5–7%.

When multinationals adopt a composite e-commerce entry model such as “E+FBP” or “E+FBP+SOP,” e-commerce platforms influence the competitive dynamics between self-operated e-commerce and FBP merchants by modifying consumer services. Concurrently, the introduction of SOP merchants may diminish consumer trust in the products of e-retailers. Therefore, as the complexity of e-commerce platform governance increases, the e-commerce market exhibits lower initial market potential for the blockchain to emerge as a “trusted governance infrastructure,” significantly enhancing the product quality and reputation of e-tailers [2,11].

The introduction of blockchain technology will incur specific technical expenditures and may substantially affect the current business model and benefit distribution of companies [12]. Blockdata’s report indicates that, as of 2021, just 27 of the top 100 enterprises by global market capitalization have fully developed and operational blockchain solutions and products. Blockchain technology will substantially influence the strategic decisions of multinational corporations and will be a critical determinant in selecting the e-commerce entry strategy for these firms.

Global brand owners are often multinational enterprises (MNEs) with established retail divisions [13], as shown by Louis Vuitton, which opened prominent flagship stores in India, Russia, China, and South Africa as early as 2005, positioning e-retailers as direct competitors to the retail divisions of MNEs. The selection of an entrance strategy for e-retailers by multinational enterprises influences not only the wholesale revenue of their manufacturing divisions but also significantly affects the direct-to-consumer sales of their retail operations. Moreover, corporate income tax significantly influences all aspects of MNEs’ decisions in their global operations [14]. The manufacturing divisions of MNEs are often situated in countries with elevated tax rates, whereas the retail divisions are positioned in areas with reduced tax rates. Newell Brands, headquartered in Hoboken, New Jersey, USA, is subject to a corporate income tax rate of around 35%. In contrast, its retail division in the Asia-Pacific area has a corporate income tax rate of around 20%. Consequently, the MNE can exploit the tax disparities between the two regions to diminish the profit of the manufacturing division situated in the high-tax area while augmenting the profit of the retail division in the low-tax area by reducing the wholesale prices of the global brand’s products, thereby achieving tax shifting and benefiting from tax planning [15]; conversely, the implementation of blockchain technology by the MNE would enhance the e-retailers reputation and intensify competitive pressure within the retail division of MNEs [16,17]. In this scenario, the MNE will elevate the wholesale prices of their products to enhance wholesale profits from e-retailers. However, due to the arm’s length principle (ALP), the pricing of products sold to their retail divisions will also be adjusted upward [18], thereby diminishing tax planning advantages and influencing the choice of e-commerce entry model by the MNE.

**Fig 1:**
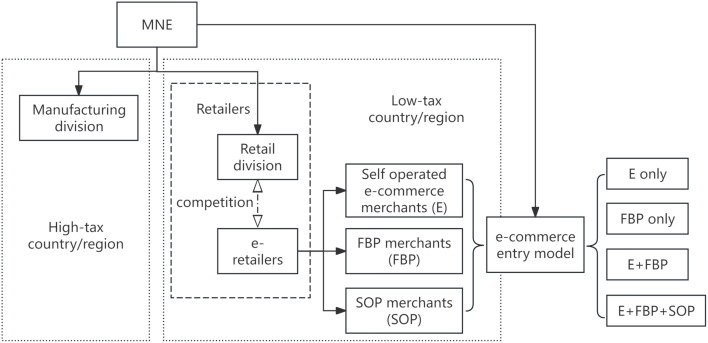
Model building.

Against the aforementioned backdrop, we develop a supply chain model for MNEs and e-retailers, incorporating the introduction of blockchain technology, customer preferences, and tax disparities. In this paper, we examine the operational dynamics of the diverse entry models of e-commerce platforms, focusing on the incorporation of third-party merchants through the Platform Opening Program (POP). It posits that e-retailers consist of self-operated e-commerce merchants, those utilizing the FBP entry model, and those employing the SOP entry model. Examine four distinct e-commerce entry models, i.e., (1) “Self-operated e-commerce exclusively”; (2) “FBP merchants exclusively”; (3) “E+FBP,” whereby e-commerce platforms and FBP merchant products coexist; and (4) “E+FBP+SOP,” where the e-commerce platform, FBP merchant, and SOP merchant cohabit. A MNE comprises a manufacturing division in a high-tax area and a retail division in a low-tax area, distributing its products in international markets via its retail division and third-party e-retailers. The model is constructed as shown in Fig 1.‌‌

Thus, the questions of interest are: (1) how can MNEs select the optimal e-commerce entry model to maximize overall profit? (2) how does introducing or not introducing blockchain technology for quality verification influence this choice? Moreover, (3) How do factors like consumer utility, preference, and tax differences impact the selection of e-commerce entry models by MNEs?

## Literature review

In this paper, we examine MNEs’ strategic decisions regarding various e-commerce entry models inside supply chain frameworks from the standpoint of blockchain technology. The paper primarily analyzes the effect mechanism of blockchain technology implementation, tax planning in international supply chains, and channel selection techniques in dual-channel supply chains.

### Impact mechanisms introduced by blockchain technology

Utilizing blockchain as a novel digital technology in supply chains has garnered significant interest from scholars. Choi (2019) [19] conducts a comparative analysis of the effects of supply chain members’ benefits through the lens of diamond identification and certification utilizing blockchain technology in the industrial sector. Mettler (2016) [20] examines the implications of blockchain technology in the pharmaceutical domain across four dimensions, i.e., public health management, user orientation, medical research, and drug counterfeiting. In the biological sector, Niu, B.Z (2021) [21] discusses the significance of blockchain in identifying the responsible party for bacterial contamination and the incentives for supply chain participants to engage. The quality of products and services significantly influences consumers’ purchasing decisions [22]; however, consumers often cannot accurately assess the quality of a product or service prior to purchase and use. In enterprise operation management, using blockchain technology for supply chain traceability offers a viable solution to address information discrepancies between suppliers and consumers [23].

To enhance consumer perceptions of quality and subsequently increase the utility of products, firms may implement blockchain technology for quality verification. This approach aims to bolster consumer confidence, reduce product uncertainty, elevate consumption utility, and optimize consumer service experience [13,24,25]. Hastig and Sodhi (2020) [26] posited that blockchain, grounded in distributed ledger technology, facilitates information transparency and product traceability within supply chains, serving as a crucial motivator for all stakeholders to adopt blockchain technology. Garaus et al. (2021) [27] found that, from the perspective of consumer choice of retailers, consumers preferred retailers utilizing a blockchain food traceability system over those employing a company’s food traceability system, with trust serving as a mediating factor. Wang et al. (2019) [28] posited that the implementation of blockchain technology may influence firm performance positively or negatively.

### Tax planning in a multinational supply chain

Globalization has led to disparities in corporate income tax rates and policies across countries, creating opportunities for MNEs to engage in tax avoidance strategies. MNEs can employ transfer pricing, tax shelters, and various strategies to minimize the tax burden of multinational supply chains in high-tax jurisdictions, thereby achieving tax optimization and enhancing tax planning advantages. A substantial body of literature has examined the influence of corporate income tax on the optimal decision-making processes of MNEs within global supply chains. Shunko et al. (2014) [29] examined the conflicting trade-offs between the incentive function of transfer pricing and the tax implications encountered by MNEs in establishing transfer prices. Xiao, WQ et al. (2015) [30] posited that the tax cross-credit policy enables MNEs to leverage excess foreign tax credits (FTCs) from subsidiaries in high-tax jurisdictions to reduce tax liabilities in low-tax divisions, thereby enhancing corporate profit optimization strategies. Shunko et al. (2017) [31] examined the establishment of subsidiaries by MNEs for product distribution in low-tax jurisdictions, focusing on allocating profits among divisions to enhance after-tax profits. Kim B et al. (2018) [32] examined the structure of MNEs, which include production and retail divisions. They analyzed the trade-offs in supply chain structure when MNEs encounter the profit-maximizing advantages of centralized operations versus the tax-saving benefits.

The tax planning decisions of MNEs will be significantly influenced by the ALP [33]. Autrey and Bova (2012) [34] examine the transfer pricing decisions of MNEs across sectors in varying tax jurisdictions, contending that a grey market may diminish domestic consumer surplus and negate any benefits derived from arm’s length transfer pricing practices. Yao (2013) [35] analyzes the impact of the arm’s length principle on multinational firms, finding that it does not enhance tax revenues when location choices are endogenous. The study concludes that transfer pricing regulation, contrary to the prevailing belief among tax authorities, actually decreases tax revenues.

### Dual-channel supply chain

The advancement of the Internet and information technology offers significant opportunities for enterprises [36,37]. An increasing number of businesses are establishing online markets through third-party e-commerce platforms, building upon traditional offline retail channels. This strategy enables them to reach a broader consumer base, gather extensive demand information, manage market pricing, enhance sales profitability, and cultivate a brand image [38, 39, 40]. Consequently, this trend stimulates research in related fields. Ryan, J.K. et al. (2012) [40] examined the equilibrium state of retailers and emerging e-commerce platforms by developing an online sales decision model. This model features a retailer selling products exclusively through its website alongside an e-commerce platform offering its products. Abhishek, V et al. (2016) [41] addressed the issue of e-commerce platforms by developing a theoretical model that encompasses agency sales and resale options. Jianxiong Zhang et al. (2017) [42] examined the effects of dynamic pricing on supply chain performance, analyzing a Stackelberg game involving a manufacturer and an online retailer to derive an equilibrium strategy. The manufacturer, as the leader, determines the advertising placement and wholesale price, whereas the online retailer, as the follower, establishes the retail price. Pu, X.J et al. (2020) [43] analyzed three online sales strategies employed by vendors, including direct sales to consumers, resale through e-retailers, and agency sales via online agents with commission payments. In this paper, they concluded that strategies implemented under lower online costs could reduce profits for traditional retailers and diminish total profits for the supply chain. Baozhuang Niu et al. (2021) [13] examined the motivations of MNEs to support e-retailers via blockchain by developing a collaborative supply chain. This supply chain includes a manufacturing division of an MNE situated in a high-tax jurisdiction, a retailing division of an MNE in a low-tax jurisdiction, and an e-retailer, focusing on issues related to product quality verification.

The ongoing evolution of the e-commerce entry model has led to the emergence of the “new retail” concept within e-commerce platforms. This diversification of entry models among e-retailers has garnered significant interest from scholars. Li, ML et al. (2020) [44] examined offline experience stores to offer experiential services for online retailers, addressing the deficiency of physical interaction in online retail. The study also investigated the impact of competition within offline experience stores. This study examines the optimal pricing decisions within the supply chain following the emergence of competitive online retailers in offline experience stores. Wang, X et al. (2019) [45] examined the optimal strategy for e-commerce platforms and suppliers. Suppliers may access the platform to sell via the FBP or SOP entry models. Tao Wang and Bo Yan (2024) [2] developed the platform by creating the “E only” or “FBP only” model, the “E+FBP” model, and the “E+FBP+SOP” model of online channel competition, analyzing participants’ pricing and service decisions across these various models.

The aforementioned literature primarily concentrates on channel selection strategies within the dual-channel supply chains of traditional enterprises, with limited scholarly attention given to the dual-channel supply chains of MNEs. Consequently, there is a paucity of research regarding the influence of tax planning strategies in the dual-channel supply chains of MNEs. Furthermore, in existing supply chain management literature, most scholars simplify the online channel in dual-channel supply chains into a single homogeneous entity or focus solely on a specific model. However, with the development of e-commerce, models featuring two or even multiple e-retailers coexisting have become mainstream. Yet few scholars have conducted in-depth research on the reality of different entry models coexisting within e-commerce platforms. This simplification overlooks fundamental differences in cost structures, service commitments, consumer trust foundations, and platform interactions across models, potentially obscuring the heterogeneous impact of new technologies like blockchain under varying channel configurations. Therefore, systematically characterizing and comparing multiple e-commerce entry models is not only essential for reflecting reality but also provides a theoretical foundation for understanding the interplay between corporate decision-making, channel competition, and technological innovation. Given this, this paper constructs an analytical framework encompassing four typical e-commerce entry modes within multinational dual-channel supply chains from a blockchain perspective. It comprehensively considers consumer utility and preferences, service sensitivity, the impact of blockchain technology, and tax differences to investigate MNE corporations’ optimal entry mode selection. This work not only expands the research boundaries of dual-channel supply chain theory but also provides more refined decision guidance for corporate channel strategies under the platform economy. The research positioning of this paper relative to relevant literature is summarized in Table 1.

**Table 1:** Comparison of research positioning and theoretical advancement between this Paper and related literature.

Author	Blockchain applications in supply Chain	Tax Planning for cross-border supply chain	Dual-channel supply chain Strategy	Analysis of e-commerce entry models
*Choi* (2019)	✓			
*Mettler* (2016)	✓			
*Hastig* & *Sodhi* (2020)	✓			
*Niu et al*. (2021)	✓	✓	✓	
*Shunko et al*. (2014)		✓		
*Xiao et al*. (2015)		✓		
*Ryan et al*. (2012)			✓	
*Abhishek et al*. (2016)			✓	
*Pu et al*. (2020)*Pu et al*. (2020)			✓	
*Wang et al*. (2019)			✓	✓
*Wang Tao and Yan Bo* (2024)			✓	✓
*Lu et al*. (2024)	✓		✓	
*Rapezzi et al*. (2025)	✓			
*This paper*	✓	✓	✓	✓

Consequently, our primary contributions are as follows.

(1) In this paper, we classify the online channel in the dual-channel supply chain as “E only,” “FBP only,” “E+FBP+SOP,” and “E+FBP+SOP.” The four distinct e-retailer entry models challenge the prior idea of one internet channel and align more closely with reality. This classification approach represents the competitive landscape under various entrance models and offers more tailored recommendations for MNEs to select the most advantageous e-commerce entry strategy.
(2) In this paper, we propose an analytical framework for MNEs operating dual-channel supply chains across heterogeneous tax jurisdictions, establishing a manufacturing division in high-tax regions and retail divisions in low-tax regions. Through a comprehensive game-theoretic perspective, we systematically examine the strategic benefits of integrated tax optimization in cross-border supply chain operations. This analytical approach advances traditional supply chain management paradigms by optimizing the strategic decisions for transfer pricing, providing empirically grounded insights for optimizing global resource allocation and enhancing multinational tax efficiency.
(3) Current research on blockchain mainly emphasizes qualitative examination of financial services, digital money, and supply chain management. This paper constructs a Stackelberg game model, derives equilibrium solutions through numerical simulation, and employs a quantitative analysis method based on mathematical modeling. It establishes comparable mathematical models before and after the implementation of blockchain technology, thereby providing a mathematical basis for theoretical deduction and strategic selection.

## Methodology

### Variables and assumptions

In this paper, we develop a two-tier supply chain model with a manufacturing division in a high-tax area and many retailers in a low-tax area. The subscript i = R, E, A, and B represents retailers. R denotes the retail division of the MNE, E signifies self-operated e-commerce, A refers to merchants utilizing the FBP entry model (hereafter termed FBP merchants), and B indicates merchants employing the SOP entry model (hereafter referred to as SOP merchants). Symbol usage throughout the text maintains the consistency described above. Decision variables and related parameters are listed in Table 2.

**Table 2:** Nomenclature.

Decisions variables	Description
*P*_*i*_	Product selling price.
*w*	Product wholesale price.
**Parameters**	**Description**
*v*	Consumer valuation of the product.
$\theta_{i}$	Consumer preference for the product.
*s*_*i*_	Level of service provided by retailers.
β	Service sensitivity factor for consumers.
τ	Tax differences between high and low tax jurisdictions.
λ	The impact of consumer preference for product valuation due to the introduction of blockchain technology.
μ	The impact of consumer preference for services due to the introduction of blockchain technology.
*F*	The cost of introducing blockchain technology for the MNE.
*x*	commission rate
*c*	Unit service fee
*f*	Franchise fee
*U*_*i*_	Consumer utility.
*D*_*i*_	Consumer demand for products.
$\pi_{F}$	Total profits of the MNE.

we make the following assumptions based on the research conducted. (1) The market is fully covered, and consumers’ valuation (v) of products follows a uniform distribution on the interval (0, 1) [46]. (2) Consumers exhibit a higher preference for MNE retail division products compared to Self-operated e-commerce products, which in turn are preferred over FBP merchant products, while FBP merchant products are favored more than SOP merchant products [8]. Without loss of generality, such that the consumer preference for MNE retail division products is 1, i.e., $0\leq \theta_{B}\leq \theta_{A}\leq \theta_{E}\leq 1$. Similarly, the relationship between the size of the level of service provided by the retailer is $0<s_{B}<s_{A}<s_{E}<s_{R}$. (3) Blockchain technology can enhance consumers’ valuation preferences and service perceptions toward e-retailers’ products, i.e., λ>1,μ>1 [10,11]. However, offline retail channels retain inherent advantages in physical experiences, meaning consumers’ upper limit of preference for online channels remains below offline channels, i.e., $\lambda \theta_{E}<1$. Moreover, the upper limit of service enhancement foronline channels remains below the offline baseline, i.e., $\mu s_{E}<s_{R}$ [47]. This translates to $1<\lambda <\frac{1}{\theta_{E}}$ and $1<\mu <\frac{s_{R}}{s_{E}}$. (4) MNEs adhere to the arm’s length principle (ALP), ensuring that the transfer price from the manufacturing division to the retail division aligns with the wholesale price of the e-retailer’s products. (5) Furthermore, MNEs can select any combination of e-commerce entry models.

**Fig 2:**
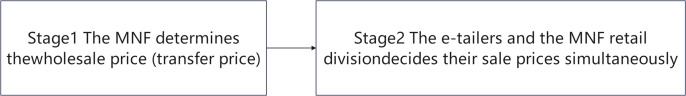
The sequence of events.

This model represents a two-stage Stackelberg game. As the leader, the MNE determines the wholesale price in the first stage, basing its decision on rational expectations of the retailers’ pricing reactions in the second stage. After observing the wholesale price set in the first stage, the MNE’s retail division and each e-retailer, acting as followers, simultaneously engage in non-cooperative Bertrand price competition to determine their respective retail prices to end consumers. Finally, we employ backward induction to solve for the subgame-perfect Nash equilibrium of this game. Fig 2 illustrates the sequence of events.

### The model

This section constructs game models for four e-commerce entry models. To clearly reveal the intrinsic logic of mode evolution, we first compare and analyze them across four key strategic dimensions, as shown in Table 3. This framework demonstrates that mode evolution is not merely a simple aggregation of retailers, but rather a systemic transformation involving the nature of competition, coordination mechanisms, and technological value. This transformation fundamentally influences subsequent equilibrium conditions.

**Table 3:** Strategic dimensions comparison of four e-commerce entry models.

Entry Model	The essence of channel conflict	Services and cost structure	Sensitivity to blockchain-enabled solutions
*E only*	The retail division of the MNE competes directly with self-operated e-commerce, where online channels maintain a unified brand and service image.	Self-operated e-commerce internalizes costs and standardizes service levels.	Low. Self-operated e-commerce already enjoys high trust endorsement, making blockchain’s marginal utility for image enhancement negligible.
*FBP only*	The MNE’s retail divisions compete with third-party FBP merchants. Online channels serve as third-party endorsements for the platform.	FBP merchants pay commissions, product service fees, and franchise fees to access e-commerce platform services, with costs being explicit.	Lower. FBP merchants already benefit from the platform’s unified quality inspection and logistics services, so the additional trust enhancement from blockchain is limited.
*E* + *FBP*	The MNE’s retail division faces competition from both self-operated e-commerce and third-party merchants, while e-retailers engage in horizontal competition within the platform. Channel conflicts have become increasingly complex.	Both self-operated e-commerce and FBP merchants provide homogeneous services through the e-commerce platform, which may influence their competitiveness by adjusting the services offered to consumers.	High. Consumers face uncertainty when choosing between “self-operated stores vs. platform-certified third parties.” Blockchain-provided unified, verifiable information can significantly reduce trust costs and resolve channel conflicts.
*E* + *FBP* + *SOP*	Building upon the self-operated + FBP model, SOP merchants introduce low-cost, low-service options. This creates a “pull-down” effect on the overall image of online channels, introducing multiple competitive dimensions.	SOP merchants pay commissions and franchise fees to access sales platforms. The tiered service structure lowers overall service expectations for online channels.	Higher. The increased autonomy of SOP merchants has heightened consumer concerns regarding product authenticity and service quality. Blockchain has emerged as the critical infrastructure for establishing a comprehensive trustworthy transaction environment and distinguishing between reputable and substandard merchants.

#### “E only” entry model.

**Fig 3:**
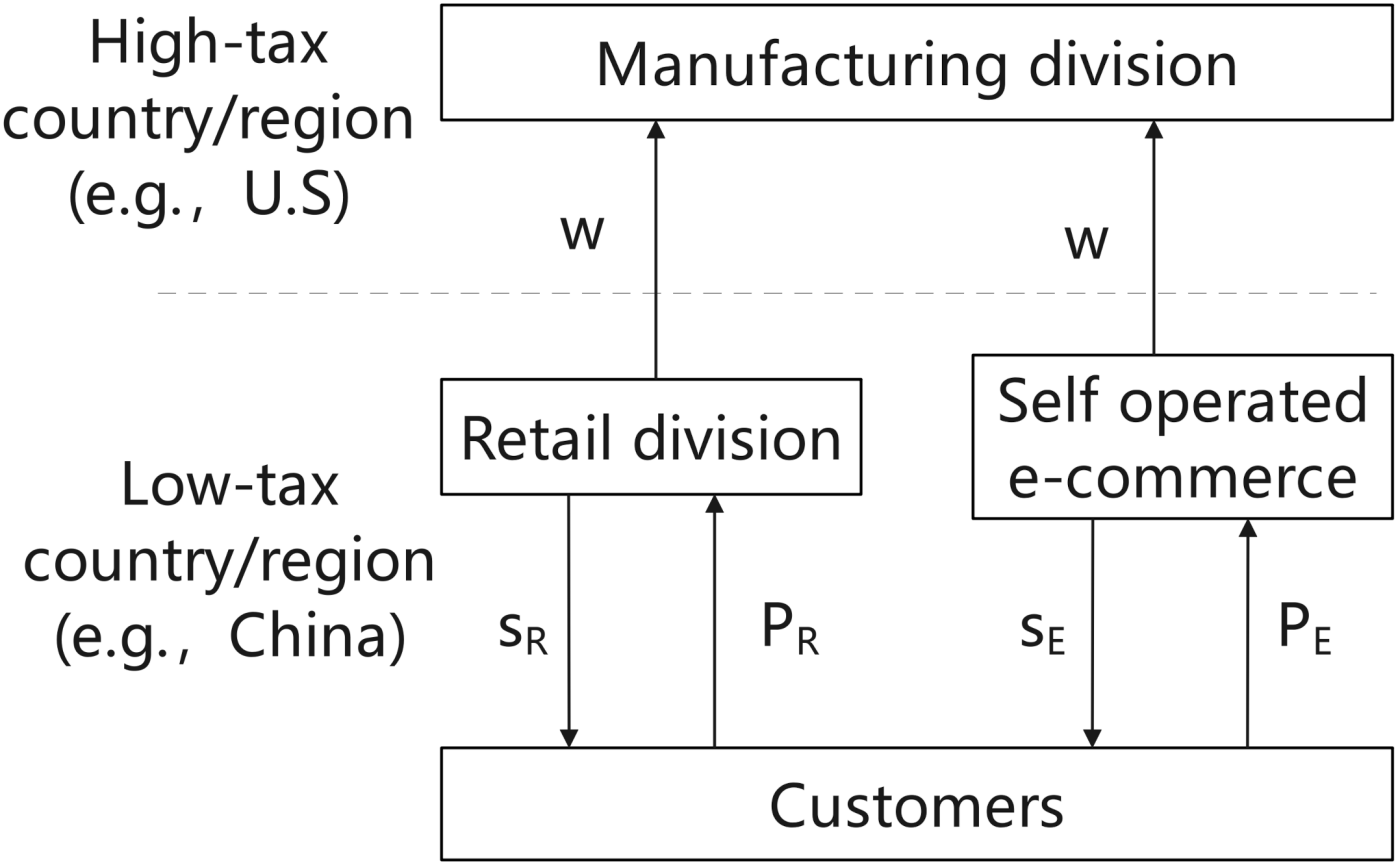
Competition for the “E only” entry model.

The model considers an e-commerce entry model in which only Self-operated e-commerce exists among the e-retailers, as shown in Fig 3, when there are two competing market sectors, Self-operated e-commerce and the MNE’s manufacturing division. Amazon, one of the largest e-commerce platforms globally, initially implemented a self-operated e-commerce model, wherein the platform procured goods and sold them directly to consumers. *w* represents the wholesale or transfer price of the product, s_i_ denotes the service rendered by the retailer during the sale to consumers, and P_i_ indicates the selling price of the product (i = E, R, A, B).

Initially, the MNE establishes the product’s wholesale price/transfer price (w) in the manufacturing division. It then sells the product at this wholesale price to both the Self-operated e-commerce and the retail division of the MNE during the wholesale stage. The retail division of the MNE and the Self-operated e-commerce division establish the product’s selling prices (*P*_*R*_ and *P*_*E*_) respectively, according to the wholesale/transfer price. In the retail phase, the Self-operated e-commerce sector offers the service (*s*_*E*_), and sells the product to the customers at a price (*P*_*E*_), whereas the retail division of the MNE provides the service (*s*_*R*_), and sells the product to the consumer at the price (*P*_*R*_). The MNE is situated in high-tax regions, but the retail division of MNEs and self-operated e-commerce are positioned in low-tax regions. As blockchain technology matures, an increasing number of MNEs will conduct quality inspections through introducing blockchain technology. Consequently, we will concentrate on the MNE’s optimum decision-making following blockchain technology’s introduction to offer complete theoretical guidance for MNEs.

With the issue and its explanation, acknowledge that customer valuation of the product positively influences utility and employ valuation preferences to distinguish the consumer value of each retailer’s goods. In this instance, customers have the highest preference for the retail division of MNE, with $\theta_{R}$ assigned a value of 1 for simplicity. The higher the selling price of the product, the more detrimental the effect on utility. The product of the service level offered by each retailer and the consumer’s service sensitivity coefficient can be viewed as the service experience received during the purchasing process, which positively influences utility. Implementing blockchain technology would enhance quality verification for e-retailers, hence positively influencing them, as demonstrated by heightened customer preference for e-retailer items in relation to improved service levels offered by e-retailers. It is posited that consumer preferences for e-retailers’ products are consistently inferior to those for the products of the MNE’s retail division and that the service level offered by e-retailers is perpetually lower than that provided by the MNE’s retail division. Consequently, the beneficial effects of blockchain technology on e-retailers will be subject to certain restrictions, constrained by $1<\lambda <\frac{1}{\theta_{E}}$ and $1<\mu <\frac{s_{R}}{s_{E}}$. Based on standard modeling approaches for analyzing vertically differentiated products in industrial organization theory [48], this paper adopts a linear additive form for modeling consumer utility. It extends the classical vertical differentiation model by incorporating a service term $\beta s_{i}$ (i = R,E,A,B). Currently, the consumer’s utility function is given by Eq (1).


$$\left\{ \begin{array}{c} U_{R}=v-P_{R}+\beta s_{R}\\ U_{E}=\lambda \theta_{E}v-P_{E}+\mu \beta s_{E} \end{array}\right.$$ (1)

A consumer will only desire a thing if his utility is favorable. Consequently, consumers will purchase the product just when *U*_*R*_ > 0, that is, $v>P_{R}-\beta s_{R}$. Simultaneously, the consumer’s value of the product v follows a uniform distribution throughout the interval (0, 1), resulting in the consumer’s demand function as Eq (2).


$$\left\{ \begin{array}{c} D_{R}=1-\frac{P_{R}-P_{E}+\beta (\mu_{SE}-s_{R})}{1-\lambda \theta_{E}}\\ {}[2ex]D_{E}=\frac{\lambda P_{R}\theta_{E}-P_{E}+\beta (\mu_{SE}-\lambda \theta_{E}s_{R})}{\lambda \theta_{E}(1-\lambda \theta_{E})} \end{array}\right.$$ (2)

The introduction of blockchain technology by MNE will result in a technology cost F, and consequently, the revenue function of each department is given by Eq (3).


$$\left\{ \begin{array}{c} \pi_{R}=(P_{R}-w)D_{R}\\ \pi_{E}=(P_{E}-w)D_{E}\\ \pi_{F}=w(D_{R}+D_{E})(1-\tau )+\pi_{R}-F \end{array}\right.$$ (3)

The overall profit of the MNE encompasses the wholesale profit from the manufacturing division and the retail profit from the retail division. The overall profit of the MNE may be articulated as $\pi_{F}={wD}_{E}\left(1-\tau \right)+P_{R}D_{R}\left(1-\tau \right)+\left(P_{R}-w\right)D_{R}\tau$. The profit of the MNE can be categorized into three components with wholesale profit ${wD}_{E}\left(1-\tau \right)$, retail profit $P_{R}D_{R}\left(1-\tau \right)$, and tax planning gain $\left(P_{R}-w\right)D_{R}\tau$ [13].

Proposition 1 If blockchain technology has been introduced, when the MNE opts for the “E only” self-operated e-commerce e-commerce entry model, the optimal wholesale price for its products is given by Eq (4).


$$w^{*}=\frac{8\theta_{E}\lambda +\theta_{E}^{2}\lambda^{2}+3\theta_{E}^{2}\lambda^{2}\tau +8\beta_{S}\mu -12\theta_{E}\lambda \tau -8\beta_{S}\tau \mu }{2\theta_{E}\lambda -16\tau +2\theta_{E}^{2}\lambda^{2}\tau -4\theta_{E}\lambda \tau +16}$$ (4)

The optimal selling price in the retail division is given by Eq (5).


$$P_{R}^{*}=-\frac{3w^{*}+2\beta s_{R}-\beta s_{E}\mu -\beta \theta_{E}\lambda s_{R}+2}{\theta_{E}\lambda -4}$$ (5)

The optimal selling price for Self-operated e-commerce is given by Eq (6).


$$P_{E}^{*}=-\frac{2w^{*}+\theta_{E}\lambda -\theta_{E}^{2}\lambda^{2}+2\beta_{S}\mu +\theta_{E}\lambda w^{*}-\beta \theta_{E}\lambda_{SR}-\beta \theta_{E}\lambda_{SE}\mu }{\theta_{E}\lambda -4}$$ (6)

The optimal total profit of the MNE is given by Eq (7).


$$\pi_{F}^{*}=w^{*}\left(\frac{P_{E}^{*}-\beta s_{E}\mu }{\theta_{E}\lambda }-1\right)(\tau -1)-\left(\frac{P_{E}^{*}-P_{R}^{*}+\beta (s_{R}-s_{E}\mu )}{\theta_{E}\lambda -1}-1\right)(P_{R}^{*}-w^{*})-F$$ (7)

Within the range where parameters satisfy economic assumptions, the aforementioned optimal strategy solution constitutes the unique subgame-perfect Nash equilibrium inner-point solution for this Stackelberg game. It can be demonstrated that at the equilibrium point, each retailer’s profit function is strictly concave with respect to its own price. Furthermore, the MNE’s total profit function is concave with respect to the wholesale price within the feasible region, satisfying the second-order conditions for optimization. Detailed proofs are provided in Appendix A1. The derivation process for each optimal solution is detailed in Appendix A2.

#### “FBP only” entry model.

**Fig 4:**
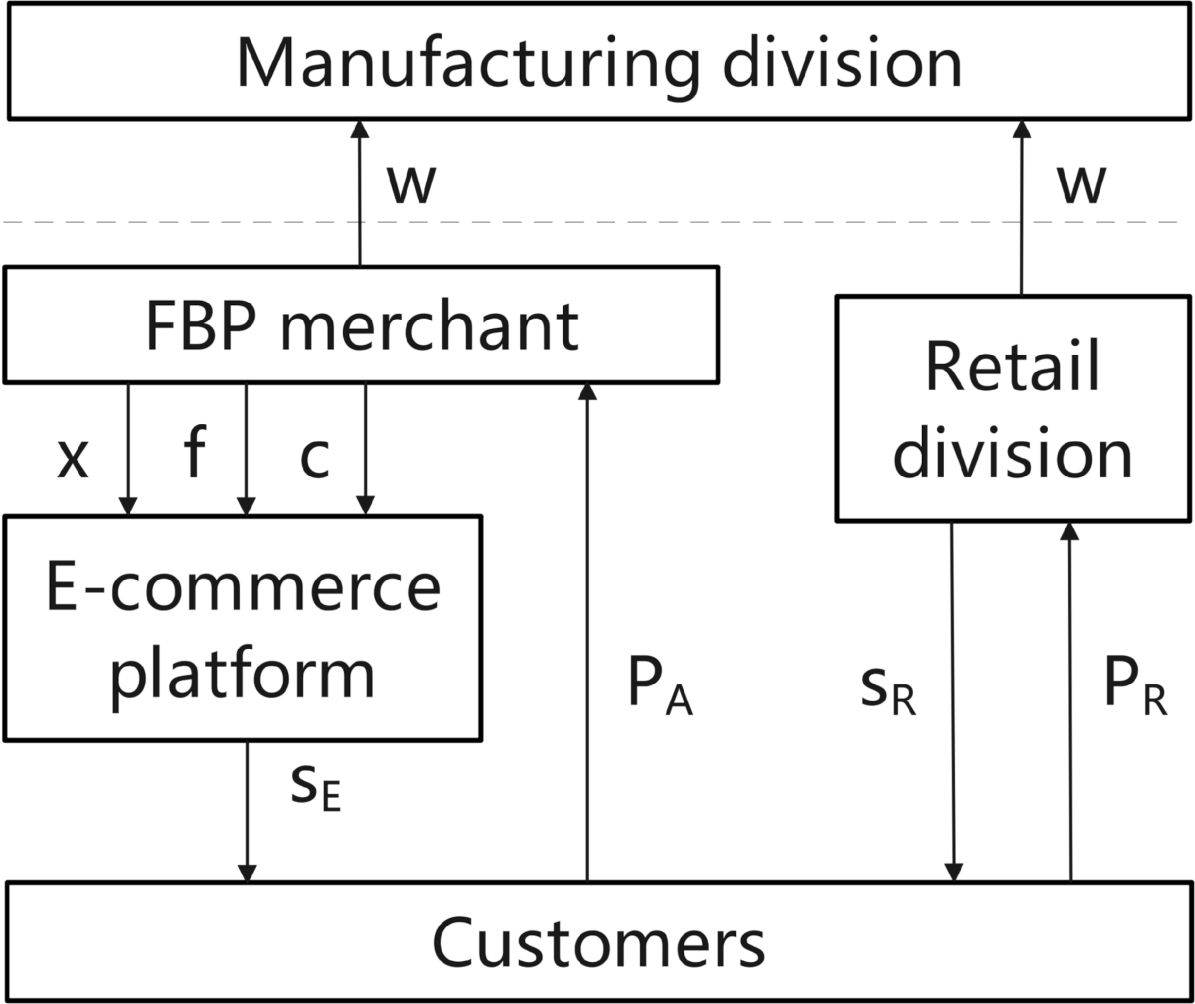
Competition for the “FBP only” entry model.

This model is structurally symmetrical to the “E only” scenario, but replaces the platform’s self-operated e-commerce operations with FBP merchants as the dominant online players. This substitution leads to two core distinctions. First, since FBP merchants must pay commissions and product service fees to the platform, their profit function differs from that of the self-operated e-commerce. This disparity in cost structures directly influences their pricing strategies. Second, consumers exhibit a lower baseline preference for FBP merchants compared to self-operated e-commerce, altering the initial competitive landscape between online channels and offline retail. In the “FBP only” e-commerce entry model, Fig 4 shows two competing market sectors of the FBP merchants and the MNE’s manufacturing division. This model is less common in reality, as MNEs usually adopt a hybrid model of “self-operated + third-party.”

Initially, the MNE establishes the product’s wholesale price/transfer price (w) in the manufacturing division. It then sells the product at this wholesale price to both the FBP merchant and the retail division of the MNE during the wholesale stage. The retail division of the MNE and the FBP merchant subsequently establish the product selling prices (*P*_*R*_ and *P*_*A*_), respectively, informed by the wholesale/transfer price. During the product retail phase, FBP merchants depend on e-commerce platforms to deliver service quality equivalent to self-operated e-commerce. They incur costs including commissions (xP_*A*_), product service fees (cD_*A*_), and franchise fees (f) while selling products to consumers at price *P*_*A*_. Meanwhile, the manufacturing division of the MNE offers service (*s*_*R*_) and sells products to consumers at price PR. The MNE’s manufacturing division is in a high-tax area, whereas the retail division and FBP merchant are in a low-tax area. In this case, the consumer utility function is given by Eq (8).


$$\left\{ \begin{array}{c} U_{R}=v-P_{R}+\beta s_{R}\\ U_{A}=\lambda \theta_{A}v-P_{A}+\mu \beta s_{E} \end{array}\right.$$ (8)

The demand function is given by Eq (9).


$$\left\{ \begin{array}{c} D_{R}=1-\frac{P_{R}-P_{A}+\beta (\mu s_{E}-s_{R})}{1-\lambda \theta_{A}}\\ {}[2ex]D_{A}=\frac{P_{R}-P_{A}+\beta (\mu s_{E}-s_{R})}{1-\lambda \theta_{A}}-\frac{P_{A}-\mu \beta s_{E}}{\lambda \theta_{E}} \end{array}\right.$$ (9)

The benefit function for each department is given by Eq (10).


$$\left\{ \begin{array}{c} \pi_{R}=(P_{R}-w)D_{R}\\ \pi_{A}=[(1-x)P_{A}-w-c]D_{A}-f\\ \pi_{F}=w(D_{R}+D_{E})(1-\tau )+\pi_{R}-F \end{array}\right.$$ (10)

The overall profit of the MNE comprises the wholesale profit from the manufacturing division and the retail profit from the retail business. The overall profit of the MNE can be articulated as ${\pi_{F}={wD}_{E}\left(1-\tau \right)+{wD}_{A}\left(1-\tau \right)+P}_{R}D_{R}\left(1-\tau \right)+\left(P_{R}-w\right)D_{R}\tau$. The profit of the MNE may be categorized into three component with wholesale profit ${wD}_{E}\left(1-\tau \right)+{wD}_{A}\left(1-\tau \right)$, retail profit $P_{R}D_{R}\left(1-\tau \right)$, and tax-planning gain $\left(P_{R}-w\right)D_{R}\tau$.

Proposition 2 When a multinational enterprise selects the e-commerce entry model of “FBP only,” and blockchain technology is implemented, the best wholesale pricing of the MNE is given by Eq (11) and Eq (12).


$$\begin{array}{c} w^{*}=-\frac{\begin{array}{cc} & \frac{\partial }{\left(\theta_{A}\lambda -4)(x-1{)}^{2}(\theta_{A}^{2}\lambda^{2}-5\theta_{A}\lambda +4\right)}\\ & +\frac{\partial }{(\theta_{A}\lambda -1)(\theta_{A}\lambda -4{)}^{2}(x-1{)}^{2}}\\ & -\frac{\left(\tau -1)(\begin{array}{cc} & 2c-3\theta_{A}\lambda -2\beta s_{E}\mu +3\theta_{A}\lambda x\\ & -\beta \theta_{A}\lambda s_{R}+2\beta s_{E}\mu x+\beta \theta_{A}\lambda s_{R}x \end{array}\right)}{\theta_{A}\lambda (\theta_{A}\lambda -4)(x-1)} \end{array}}{\begin{array}{cc} & \frac{2(2x+\theta_{A}\lambda -\theta_{A}\lambda x-1{)}^{2}}{(\theta_{A}\lambda -1)(\theta_{A}\lambda -4{)}^{2}(x-1{)}^{2}}-\frac{2(\tau -1)(\theta_{A}\lambda -\theta_{A}\lambda x+2)}{\theta_{A}\lambda (\theta_{A}\lambda -4)(x-1)} \end{array}} \end{array}$$ (11)

$$\partial =(2x+\theta_{A}\lambda -\theta_{A}\lambda x-1)(\begin{array}{c} c-2x+2\beta s_{R}-2\theta_{A}\lambda \\ -\beta s_{E}\mu -2\beta s_{R}x\\ +2\theta_{A}\lambda x-\beta_{0}\lambda s_{R}\\ +\beta s_{E}\mu x+\beta_{0}\lambda s_{R}x+2 \end{array})$$ (12)

The optimal product selling price in the manufacturing division is given by Eq (13).


$$P_{R}^{*}=\frac{c+3w^{*}-2x+2\beta s_{R}-2\theta_{A}\lambda -2w^{*}x-\beta s_{E}\mu }{-2\beta s_{R}x+2\theta_{A}\lambda x-\beta \theta_{A}\lambda s_{R}+\beta s_{E}\mu x+\beta \theta_{A}\lambda s_{R}x+2(\theta_{A}\lambda -4)(x-1)}$$ (13)

The optimal product selling price for FBP merchants is given by Eq 14.


$$P_{A}^{*}=\frac{2c+2w^{*}+\theta_{A}\lambda -\theta_{A}^{2}\lambda^{2}+\theta_{A}^{2}\lambda^{2}x+2\beta s_{E}\mu +\theta_{A}\lambda w^{*}-\theta_{A}\lambda x}{-\beta \theta_{A}\lambda s_{R}-2\beta s_{E}\mu x-\theta_{A}\lambda w^{*}x-\beta \theta_{A}\lambda s_{E}\mu +\beta \theta_{A}\lambda s_{R}x+\beta \theta_{A}\lambda s_{E}\mu x(\theta_{A}\lambda -4)(x-1)}$$ (14)

The optimal total profit of the MNE is given by Eq (15).


$$\pi_{F}^{*}=w^{*}\left(\frac{P_{A}^{*}-\beta s_{E}\mu }{\theta_{A}\lambda }-1\right)(\tau -1)-\left(\frac{P_{A}^{*}-P_{R}^{*}+\beta (s_{R}-s_{E}\mu )}{\theta_{A}\lambda -1}-1\right)(P_{R}^{*}-w^{*})-F$$ (15)

#### “E+FBP” entry model.

**Fig 5:**
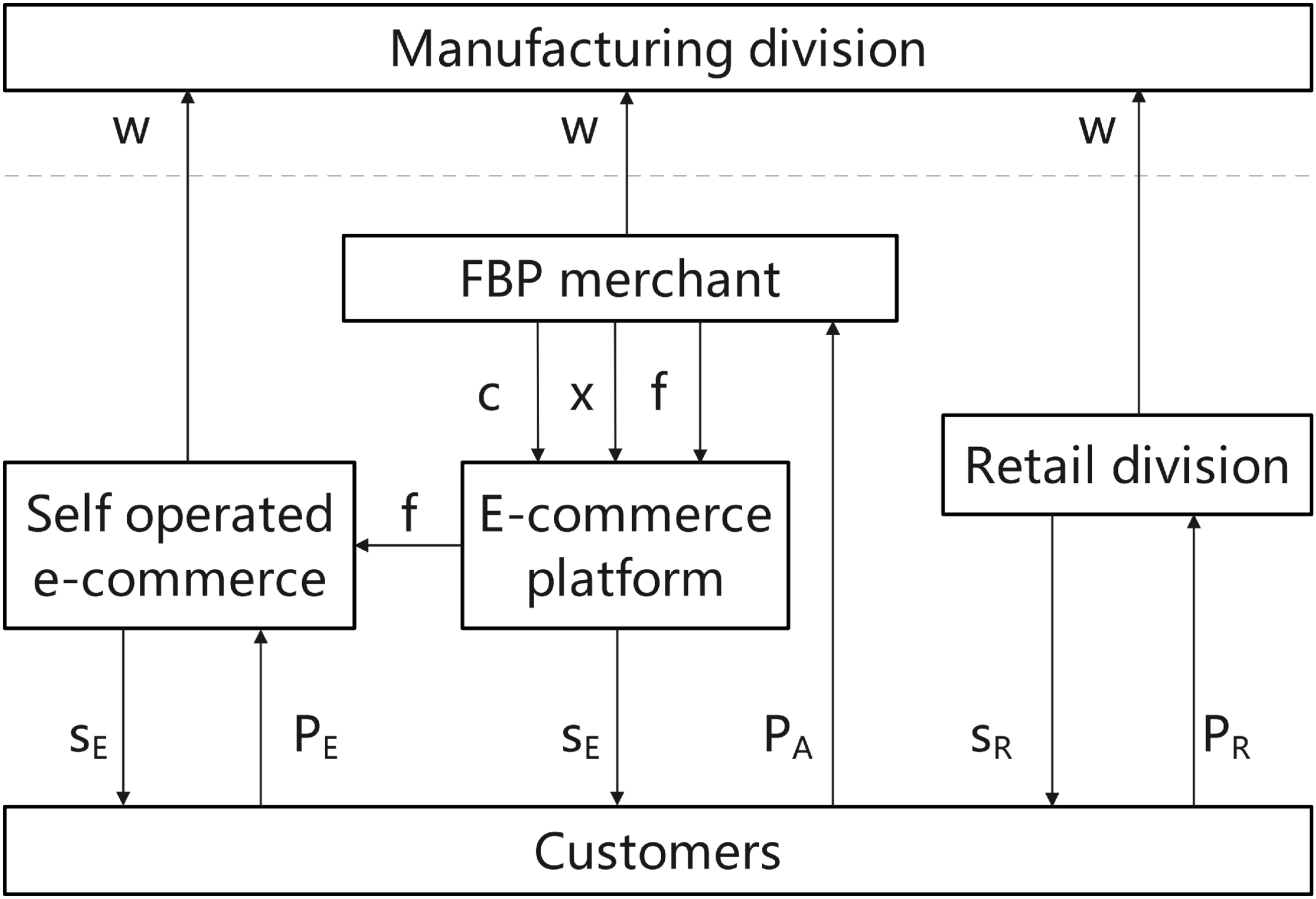
Competition for the “E + FBP” entry model.

This model introduces FBP merchants on the foundation of “E only.” This expansion introduces three new layers of complexity. First, market demand undergoes a two-stage segmentation, with consumers choosing among three retailers. Second, MNE’s wholesale profits originate simultaneously from both self-operated e-commerce and FBP merchants. Most critically, both self-operated e-commerce and FBP merchants receive identical platform services, creating competition between the platform’s self-operated operations and third-party business. Under the “E+FBP” e-commerce entry model, the market now features three competing entities: self-operated e-commerce, FBP merchants, and the MNE’s retail division. Seen in Fig 5, comprises three competing entities in the market, i.e., self-operated e-commerce, FBP merchants, and manufacturing divisions of the MNE.

The MNE first determines the wholesale price/transfer price (w) for the product from the manufacturing division. It then sells this product to the self-operated e-commerce platform, FBP merchant, and the MNE’s retail division at the established wholesale price or transfer price during the wholesale stage. During the product retailing phase, the FBP merchant utilizes the e-commerce platform to deliver services consistent with self-operated e-commerce. The FBP merchant compensates the e-commerce platform with a commission of xP_*A*_, a product service fee of cD_*A*_, and a joining fee of f while selling the product to consumers at a price *P*_*A*_. In contrast, the self-operated e-commerce offers services *s*_*E*_ and retails the product to consumers at a price *P*_*E*_, also incurring a joining fee of f from the e-commerce platform for the FBP merchant. Additionally, the manufacturing division of the MNE provides services *s*_*R*_ and sells products to consumers at a price *P*_*R*_. The manufacturing division of the MNE is situated in high-tax regions. Meanwhile, the retail division, the self-operated e-commerce, and FBP merchants are positioned in low-tax regions (*P*_*A*_), product service fees (cD_*A*_). The description and assumptions indicate that the “E+FBP” mode consists of the “E only” mode, followed by adding FBP merchants. The consumer’s utility function at this stage is given by Eq (16).


$$\left\{ \begin{array}{c} U_{R}=v-P_{R}+\beta s_{R}\\ U_{E}=\lambda \theta_{E}v-P_{E}+\mu \beta s_{E}\\ U_{A}=\lambda \theta_{A}v-P_{A}+\mu \beta s_{E} \end{array}\right.$$ (16)

Consumers concurrently require products from the manufacturing division, self-operated e-commerce, and FBP merchants, with the consumer demand function given by Eq (17).


$$\left\{ \begin{array}{c} D_{R}=1-\frac{P_{R}-P_{E}+\beta (\mu s_{E}-s_{R})}{1-\lambda \theta_{E}}\\ {}[2ex]D_{E}=\frac{P_{R}-P_{E}+\beta (\mu s_{E}-s_{R})}{1-\lambda \theta_{E}}-\frac{P_{E}-P_{A}}{\lambda (\theta_{E}-\theta_{A})}\\ {}[2ex]D_{A}=\frac{P_{E}-P_{A}}{\lambda (\theta_{E}-\theta_{A})}-\frac{P_{A}-\mu \beta s_{E}}{\lambda \theta_{A}} \end{array}\right.$$ (17)

The implementation of blockchain technology by the MNE will result in a technology cost of F. Thus, the benefit function for each department is given by Eq (18).


$$\left\{ \begin{array}{c} \pi_{R}=(P_{R}-w)D_{R}\\ \pi_{E}=(P_{E}-w)D_{E}+f\\ \pi_{A}=[(1-x)P_{A}-w-c]D_{A}-f\\ \pi_{F}=w(D_{R}+D_{E}+D_{A})(1-\tau )+\pi_{R}-F \end{array}\right.$$ (18)

The overall profit of the MNE comprises the wholesale profit from the manufacturing division and the retail profit from the retail business. The total profit of the MNE may be articulated as$\pi_{F}={wD}_{E}\left(1-\tau \right)+{wD}_{A}\left(1-\tau \right)+P_{R}D_{R}\left(1-\tau \right)+\left(P_{R}-w\right)D_{R}\tau$. The MNE’s earnings may be categorized into three components, namely, wholesale profits ${wD}_{E}\left(1-\tau \right)+{wD}_{A}\left(1-\tau \right)$, retail profits $P_{R}D_{R}\left(1-\tau \right)$, and tax planning gains $\left(P_{R}-w\right)D_{R}\tau$.

Proposition 3 When a multinational enterprise selects the e-commerce entry model of “E+FBP,” and blockchain technology is implemented, the optimal wholesale pricing of the MNE’s products is given by Eq (19).


$$\begin{array}{c} w^{*}=-\frac{\begin{array}{cc} & \frac{(\theta_{A}-\theta_{E}-\theta_{A}x+2\theta_{E}x)\left(\begin{array}{cc} & \theta_{A}-4\theta_{E}-c\theta_{E}-\theta_{A}x+4\theta_{E}x+4\theta_{E}^{2}\lambda -4\theta_{E}^{2}\lambda x\\ & \;-3\theta_{A}\theta_{E}^{2}\lambda^{2}+\beta \theta_{A}s_{R}-4\beta \theta_{E}s_{R}+2\theta_{A}\theta_{E}\lambda +c\theta_{E}^{2}\lambda \\ & \;+3\beta \theta_{E}s_{E}\mu -\beta \theta_{A}s_{R}x+4\beta \theta_{E}s_{R}x-2\theta_{A}\theta_{E}\lambda x\\ & \;+2\beta \theta_{E}^{2}\lambda s_{R}+3\theta_{A}\theta_{E}^{2}\lambda^{2}x+\beta \theta_{A}\theta_{E}\lambda s_{R}-3\beta \theta_{E}s_{E}\mu x\\ & \;-\beta \theta_{E}^{2}\lambda s_{E}\mu -2\beta \theta_{E}^{2}\lambda s_{R}x+\beta \theta_{E}^{2}\lambda s_{E}\mu x\\ & \;-2\beta \theta_{A}\theta_{E}\lambda s_{E}\mu -\beta \theta_{A}\theta_{E}\lambda s_{R}x+2\beta \theta_{A}\theta_{E}\lambda s_{E}\mu x \end{array}\right)}{2(x-1{)}^{2}(\theta_{A}-4\theta_{E}+\theta_{E}^{2}\lambda +2\theta_{A}\theta_{E}\lambda {)}^{2}}\\ \left[4pt\right] & +\frac{(\tau -1)\left(\begin{array}{cc} & \theta_{A}^{2}\lambda x-\theta_{A}^{2}\lambda -4c\theta_{E}-\theta_{A}\theta_{E}^{2}\lambda -5\theta_{A}^{2}\theta_{E}\lambda^{2}+7\theta_{A}\theta_{E}\lambda +c\theta_{E}^{2}\lambda \\ & \;+3c\theta_{A}\theta_{E}\lambda +2\beta \theta_{A}s_{E}\mu +4\beta \theta_{E}s_{E}\mu -7\theta_{A}\theta_{E}\lambda x-\beta \theta_{A}^{2}\lambda s_{R}+\theta_{A}\theta_{E}^{2}\lambda x\\ & \;+5\theta_{A}^{2}\theta_{E}\lambda^{2}x+\beta \theta_{A}\theta_{E}\lambda s_{R}-2\beta \theta_{A}s_{E}\mu x-4\beta \theta_{E}s_{E}\mu x-2\beta \theta_{A}^{2}\lambda s_{E}\mu \\ & \;-\beta \theta_{E}^{2}\lambda s_{E}\mu +\beta \theta_{A}^{2}\lambda s_{R}x+2\beta \theta_{A}^{2}\lambda s_{E}\mu x+\beta \theta_{E}^{2}\lambda s_{E}\mu x-3\beta \theta_{A}\theta_{E}\lambda s_{E}\mu \\ & \;-\beta \theta_{A}\theta_{E}\lambda s_{R}x+3\beta \theta_{A}\theta_{E}\lambda s_{E}\mu x \end{array}\right)}{2\theta_{A}\lambda (x-1)(\theta_{A}-4\theta_{E}+\theta_{E}^{2}\lambda +2\theta_{A}\theta_{E}\lambda )} \end{array}}{\begin{array}{cc} & \frac{(\theta_{E}\lambda -1)(\theta_{A}-\theta_{E}-\theta_{A}x+2\theta_{E}x{)}^{2}}{2(x-1{)}^{2}(\theta_{A}-4\theta_{E}+\theta_{E}^{2}\lambda +2\theta_{A}\theta_{E}\lambda {)}^{2}}\\ \left[4pt\right] & +\frac{(\tau -1)(\begin{array}{cc} & 2\theta_{A}x-4\theta_{E}-2\theta_{A}+3\theta_{A}^{2}\lambda \\ & +\theta_{E}^{2}\lambda -3\theta_{A}^{2}\lambda x+2\theta_{A}\theta_{E}\lambda +\theta_{A}\theta_{E}\lambda x \end{array}}{\theta_{A}\lambda (x-1)(\theta_{A}-4\theta_{E}+\theta_{E}^{2}\lambda +2\theta_{A}\theta_{E}\lambda )} \end{array}} \end{array}$$ (19)

The optimal product selling price in the manufacturing division is given by Eq (20).


$$\begin{array}{c} P_{R}^{*}=-\frac{\begin{array}{cc} & \theta_{A}-4\theta_{E}-c\theta_{E}+\theta_{A}w^{*}-7\theta_{E}w^{*}-\theta_{A}x+4\theta_{E}x+4\theta_{E}^{2}\lambda +\theta_{E}^{2}\lambda w^{*}-4\theta_{E}^{2}\lambda x\\ & -3\theta_{A}\theta_{E}^{2}\lambda^{2}+\beta \theta_{A}s_{R}-4\beta \theta_{E}s_{R}+2\theta_{A}\theta_{E}\lambda -\theta_{A}w^{*}x+6\theta_{E}w^{*}x+c\theta_{E}^{2}\lambda \\ & +3\beta \theta_{E}s_{E}\mu -\beta \theta_{A}s_{R}x+4\beta \theta_{E}s_{R}x+5\theta_{A}\theta_{E}\lambda w^{*}-2\theta_{A}\theta_{E}\lambda x+2\beta \theta_{E}^{2}\lambda s_{R}\\ & +3\theta_{A}\theta_{E}^{2}\lambda^{2}x+\beta \theta_{A}\theta_{E}\lambda s_{R}-3\beta \theta_{E}s_{E}\mu x-5\theta_{A}\theta_{E}\lambda w^{*}x-\beta \theta_{E}^{2}\lambda s_{E}\mu \\ & -2\beta \theta_{E}^{2}\lambda s_{R}x+\beta \theta_{E}^{2}\lambda s_{E}\mu x-2\beta \theta_{A}\theta_{E}\lambda s_{E}\mu -\beta \theta_{A}\theta_{E}\lambda s_{R}x+2\beta \theta_{A}\theta_{E}\lambda s_{E}\mu x \end{array}}{2(x-1)(\theta_{A}-4\theta_{E}+\theta_{E}^{2}\lambda +2\theta_{A}\theta_{E}\lambda )} \end{array}$$ (20)

The optimal product selling price for Self-operated e-commerce is given by Eq (21).


$$\begin{array}{c} P_{E}^{*}=\frac{\begin{array}{cc} & \theta_{E}^{3}\lambda^{2}-3\theta_{E}w^{*}-\theta_{E}^{2}\lambda -c\theta_{E}+\theta_{E}^{2}\lambda x-\theta_{A}\theta_{E}^{2}\lambda^{2}-\theta_{E}^{3}\lambda^{2}x+\theta_{A}\theta_{E}\lambda +2\theta_{E}w^{*}x\\ & +c\theta_{E}^{2}\lambda +\beta \theta_{A}s_{E}\mu -\beta \theta_{E}s_{E}\mu +3\theta_{A}\theta_{E}\lambda w^{*}-\theta_{A}\theta_{E}\lambda x+\beta \theta_{E}^{2}\lambda s_{R}+\theta_{E}^{2}\lambda w^{*}x+\theta_{A}\theta_{E}^{2}\lambda^{2}x\\ & -\beta \theta_{A}\theta_{E}\lambda s_{R}-\beta \theta_{A}s_{E}\mu x+\beta \theta_{E}s_{E}\mu x-3\theta_{A}\theta_{E}\lambda w^{*}x-\beta \theta_{E}^{2}\lambda s_{R}x+\beta \theta_{A}\theta_{E}\lambda s_{R}x \end{array}}{\left(x-1)(\theta_{A}-4\theta_{E}+\theta_{E}^{2}\lambda +2\theta_{A}\theta_{E}\lambda \right)} \end{array}$$ (21)

The optimal product selling price for FBP merchants is given by Eq (22).


$$\begin{array}{c} P_{A}^{*}=-\frac{\begin{array}{cc} & \theta_{A}^{2}\lambda -2\theta_{A}w^{*}-4\theta_{E}w^{*}-4c\theta_{E}+3\theta_{A}^{2}\lambda w^{*}+\theta_{E}^{2}\lambda w^{*}-\theta_{A}^{2}\lambda x+\theta_{A}\theta_{E}^{2}\lambda^{2}-\theta_{A}^{2}\theta_{E}\lambda^{2}\\ & -\theta_{A}\theta_{E}\lambda +2\theta_{A}w^{*}x+c\theta_{E}^{2}\lambda +3c\theta_{A}\theta_{E}\lambda +4\beta \theta_{A}s_{E}\mu -4\beta \theta_{E}s_{E}\mu +2\theta_{A}\theta_{E}\lambda w^{*}\\ & +\theta_{A}\theta_{E}\lambda x-\beta \theta_{A}^{2}\lambda s_{R}-3\theta_{A}^{2}\lambda w^{*}x-\theta_{A}\theta_{E}^{2}\lambda^{2}x+\theta_{A}^{2}\theta_{E}\lambda^{2}x+\beta \theta_{A}\theta_{E}\lambda s_{R}\\ & -4\beta \theta_{A}s_{E}\mu x+4\beta \theta_{E}s_{E}\mu x+\theta_{A}\theta_{E}\lambda w^{*}x-2\beta \theta_{A}^{2}\lambda s_{E}\mu +\beta \theta_{E}^{2}\lambda s_{E}\mu +\beta \theta_{A}^{2}\lambda s_{R}x\\ & +2\beta \theta_{A}^{2}\lambda s_{E}\mu x-\beta \theta_{E}^{2}\lambda s_{E}\mu x+\beta \theta_{A}\theta_{E}\lambda s_{E}\mu -\beta \theta_{A}\theta_{E}\lambda s_{R}x-\beta \theta_{A}\theta_{E}\lambda s_{E}\mu x \end{array}}{2(x-1)(\theta_{A}-4\theta_{E}+\theta_{E}^{2}\lambda +2\theta_{A}\theta_{E}\lambda )} \end{array}$$ (22)

The optimal total profit of the MNE is given by Eq (23).


$$\pi_{F}^{*}=w^{*}\left(\frac{P_{A}^{*}-\beta s_{E}\mu }{\theta_{A}\lambda }-1\right)(\tau -1)-\left(\frac{P_{E}^{*}-P_{R}^{*}+\beta (s_{R}-s_{E}\mu )}{\theta_{E}\lambda -1}-1\right)(P_{R}^{*}-w^{*})-F$$ (23)

#### “E+FBP+SOP” entry model.

**Fig 6:**
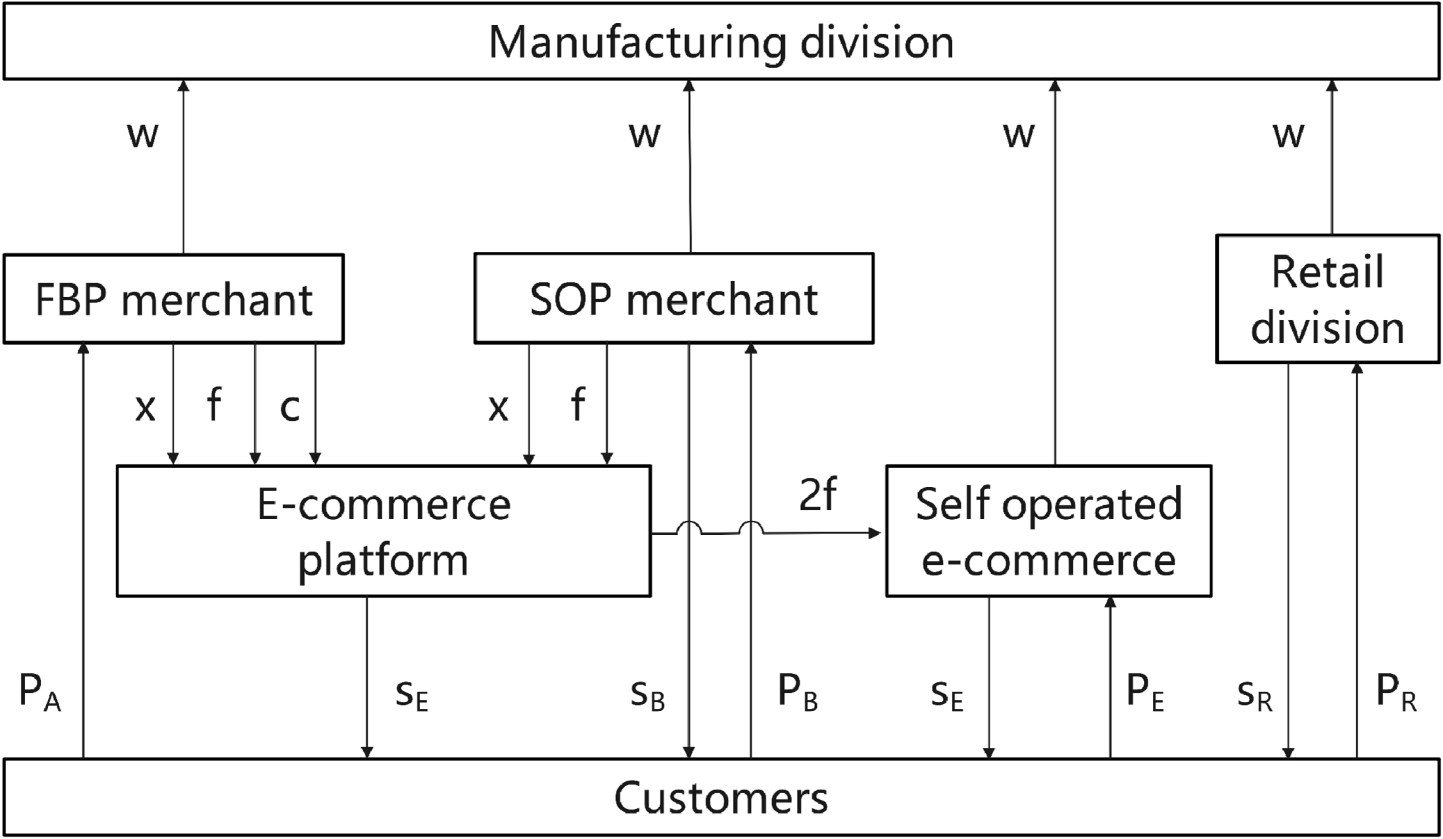
Competition for the “E+FBP+SOP” entry model.

This model builds upon the “E + FBP” e-commerce entry model by introducing independently operated SOP merchants. SOP merchants enter the market with lower baseline service levels and consumer preferences. This not only adds a new competitive dimension but also risks lowering consumers’ average quality and service expectations for the entire online channel, thereby exerting downward pressure on all online participants. Consumer demand undergoes three consecutive divisions among four retailers, further segmenting the market and dispersing MNE wholesale profit sources. Blockchain technology’s enabling value is most prominent in this model, as it effectively mitigates additional trust concerns introduced by SOP merchants, enhancing the credibility of the entire online ecosystem. As shown in Fig 6, there are four competitors, i.e., self-operated e-commerce, FBP merchants, and MNE’s manufacturing division. The MNE establishes the wholesale price or transfer price (w) for the manufacturing division’s product and subsequently sells this product to the Self-operated e-commerce, FBP merchant, SOP merchant, and the MNE’s retail division at the determined wholesale price or transfer price *w* during the wholesale stage. The retail division of the MNE, along with self-operated e-commerce, FBP merchants, and SOP merchants, concurrently establishes the selling prices of the products (*P*_*R*_, *P*_*E*_, *P*_*A*_, and *P*_*B*_), based on the wholesale or transfer price. At the retail stage, FBP merchants depend on the e-commerce platform to deliver service quality comparable to self-operated e-commerce merchants while also paying commissions to the platform. xP_*A*_ represents product service fees, cD_*A*_ denotes additional charges, and f indicates franchise fees, with products sold to consumers at *P*_*A*_ prices. SOP merchants require the e-commerce platform solely for sales, managing all services independently, thus paying only commission xP_*B*_ and franchise fee f while selling products at *P*_*B*_ prices. Self-operated e-commerce merchants offer services *s*_*E*_ and sell products at *P*_*E*_ prices, concurrently receiving a franchise fee of 2f from the e-commerce platform for FBP merchants and SOP merchants. The manufacturing division offers service *s*_*R*_ and sells the product to consumers at price *P*_*R*_. The manufacturing division of the MNE is situated in a high-tax region, whereas the retail division, self-operated e-commerce, FBP merchants, and SOP merchants are located in a low-tax region.

The “E+FBP+SOP” model is derived from the “E+FBP” model, with the addition of SOP merchants. The consumer utility function is defined as Eq (24).


$$\left\{ \begin{array}{c} U_{R}=v-P_{R}+\beta s_{R}\\ U_{E}=\lambda \theta_{E}v-P_{E}+\mu \beta s_{E}\\ U_{A}=\lambda \theta_{A}v-P_{A}+\mu \beta s_{E}\\ U_{B}=\lambda \theta_{B}v-P_{B}+\mu \beta s_{B} \end{array}\right.$$ (24)

The consumer’s demand function is given by Eq (25).


$$\left\{ \begin{array}{c} D_{R}=1-\frac{P_{R}-P_{E}+\beta (\mu s_{E}-s_{R})}{1-\lambda \theta_{E}}\\ {}[2ex]D_{E}=\frac{P_{R}-P_{E}+\beta (\mu s_{E}-s_{R})}{1-\lambda \theta_{E}}-\frac{P_{E}-P_{A}}{\lambda (\theta_{E}-\theta_{A})}\\ {}[2ex]D_{A}=\frac{P_{E}-P_{A}}{\lambda (\theta_{E}-\theta_{A})}-\frac{P_{A}-P_{B}+\mu \beta (s_{B}-s_{E})}{\lambda (\theta_{A}-\theta_{B})}\\ {}[2ex]D_{B}=\frac{P_{A}-P_{B}+\mu \beta (s_{B}-s_{E})}{\lambda (\theta_{A}-\theta_{B})}-\frac{P_{B}-\mu \beta s_{B}}{\lambda \theta_{B}} \end{array}\right.$$ (25)

The benefit function for each department is given by Eq (26).


$$\left\{ \begin{array}{c} \pi_{R}=(P_{R}-w)D_{R}\\ \pi_{E}=(P_{E}-w)D_{E}+2f\\ \pi_{A}=[(1-x)P_{A}-w-c]D_{A}-f\\ \pi_{B}=[(1-x)P_{B}-w]D_{B}-f\\ \pi_{F}=w(D_{R}+D_{E}+D_{A}+D_{B})(1-\tau )+\pi_{R}-F \end{array}\right.$$ (26)

The overall profit of the MNE comprises the wholesale profit from the manufacturing division and the retail profit from the retail business. The total profit of the MNE can be articulated as $\pi_{F}=wD_{E}\left(1-\tau \right)+wD_{A}\left(1-\tau \right)+wD_{B}\left(1-\tau \right)+P_{R}D_{R}\left(1-\tau \right)+\left(P_{R}-w\right)D_{R}\tau$. The profits of the MNE may be categorized into three components, with wholesale profits $wD_{E}\left(1-\tau \right)+wD_{A}\left(1-\tau \right)+wD_{B}\left(1-\tau \right)$, retail profit $P_{R}D_{R}\left(1-\tau \right)$, and tax planning gain $\left(P_{R}-w\right)D_{R}\tau$.

Proposition 4 When a multinational enterprise selects the e-commerce entry model of “E+FBP+SOP,” if blockchain technology has been introduced, the optimal wholesale price of the products of MNE satisfies Eq (27).


$$\left(\frac{P_{B}^{*}-\beta s_{B}\mu }{\theta_{B}\lambda }-1\right)(\tau -1)-\frac{P_{E}^{*}-P_{R}^{*}+\beta (s_{R}-\mu s_{E})}{\theta_{E}\lambda -1}-1=0$$ (27)

The optimal selling prices for the manufacturing division *P*_*R*_, the self-operated e-commerce *P*_*E*_, the FBP merchant *P*_*A*_, and the SOP merchant *P*_*B*_ concurrently fulfill Eq (28), Eq (29), Eq (30), and Eq (31).


$$\frac{\left(P_{R}^{*}-w^{*}\right)}{\theta_{E}\lambda -1}+\frac{P_{R}^{*}-P_{E}^{*}+\beta (s_{R}-\mu s_{E})+P_{R}^{*}-w^{*}}{\theta_{E}\lambda -1}+1=0$$ (28)

$$\left(\frac{1}{\theta_{A}\lambda -\theta_{E}\lambda }+\frac{1}{\theta_{E}\lambda -1}\right)(P_{E}^{*}-w^{*})-\frac{P_{A}^{*}-P_{E}^{*}}{\theta_{A}\lambda -\theta_{E}\lambda }-\frac{P_{R}^{*}-P_{E}^{*}+\beta (\mu s_{E}-s_{R})}{\theta_{E}\lambda -1}=0$$ (29)

$$-\left(\frac{1}{\lambda (\theta_{A}-\theta_{E})}+\frac{1}{\lambda (\theta_{B}-\theta_{E})}\right)(c-P_{A}^{*}+w^{*}+P_{A}^{*}x)-\left(\frac{P_{A}^{*}-P_{B}^{*}+\mu \beta (s_{B}-s_{E})}{\lambda (\theta_{B}-\theta_{E})}+\frac{P_{A}^{*}-P_{E}^{*}}{\lambda (\theta_{A}-\theta_{E})}\right)(x-1)=0$$ (30)

$$\frac{\left(x-1)(\theta_{B}P_{A}^{*}-\theta_{E}P_{B}^{*}-\mu \beta (\theta_{B}s_{E}-\theta_{E}s_{B})\right)}{\theta_{B}\lambda (\theta_{B}-\theta_{E})}-\frac{\theta_{E}(w^{*}-P_{B}^{*}+P_{B}^{*}x)}{\theta_{B}\lambda (\theta_{B}-\theta_{E})}=0$$ (31)

The optimal total profit of the MNE is given by Eq (32).


$$\pi_{F}^{*}=\left(\frac{P_{R}^{*}-P_{E}^{*}+\beta (\mu s_{E}-s_{R})}{\theta_{E}\lambda -1}+1\right)(P_{R}^{*}-w^{*})+w^{*}\left(\frac{P_{B}^{*}-\beta s_{B}\mu }{\theta_{B}\lambda }-1\right)(\tau -1)$$ (32)

When λ=μ=1 and F = 0, we can determine the optimal decisions for the wholesale price of products, the optimal pricing decisions for the retail division and e-retailers, and the optimal total profit for the MNE across all modes without introducing blockchain technology. At this point, the comparative static analysis results for key parameters without blockchain technology are shown in Table 4.

**Table 4:** Comparative static analysis of equilibrium outcomes without blockchain technology introduced.

Parameter	E only			FBP only			E + FBP			E + FBP + SOP		
	*w* ^*^	$\overset{*}{\underset{R}{P}}$	$\overset{*}{\underset{F}{\pi }}$	*w* ^*^	$\overset{*}{\underset{R}{P}}$	$\overset{*}{\underset{F}{\pi }}$	*w* ^*^	$\overset{*}{\underset{R}{P}}$	$\overset{*}{\underset{F}{\pi }}$	*w* ^*^	$\overset{*}{\underset{R}{P}}$	$\overset{*}{\underset{F}{\pi }}$
$\theta_{E}$	↑	↓	↓	–	–	–	↑	↓	↓	↓	↓	↓
$\theta_{A}$	–	–	–	↑	↓	↓	↑	↑	↓	↑	↑	↑
$\theta_{B}$	–	–	–	–	–	–	–	–	–	↑	↑	↓
*s*_*R*_	↓	↑	↑	↑	↑	↑	↑	↑	↑	↑	↑	↑
*s*_*E*_	↑	↓	↓	↑	↑	↓	↑	↓	↓	↑	↓	↓
*s*_*B*_	–	–	–	–	–	–	–	–	–	↑	↑	↑
β	↑	↑	↑	↑	↑	↑	↑	↑	↑	↑	↑	↑
τ	↓	↓	↓	↓	↓	↓	↓	↓	↓	↑	↑	↓

With blockchain technology being introduced, the comparative static analysis results for key parameters are shown in Table 5.

**Table 5:** Comparative static analysis of equilibrium outcomes with blockchain technology introduced.

Parameter	E only			FBP only			E + FBP			E + FBP + SOP		
	*w* ^*^	$\overset{*}{\underset{R}{P}}$	$\overset{*}{\underset{F}{\pi }}$	*w* ^*^	$\overset{*}{\underset{R}{P}}$	$\overset{*}{\underset{F}{\pi }}$	*w* ^*^	$\overset{*}{\underset{R}{P}}$	$\overset{*}{\underset{F}{\pi }}$	*w* ^*^	$\overset{*}{\underset{R}{P}}$	$\overset{*}{\underset{F}{\pi }}$
$\theta_{E}$	↑	↓	↓	–	–	–	↑	↓	↓	↓	↓	↓
$\theta_{A}$	–	–	–	↑	↓	↑	↑	↓	↓	↑	↑	↑
$\theta_{B}$	–	–	–	–	–	–	–	–	–	↑	↓	↓
*s*_*R*_	↑	↑	↓	↑	↑	↓	↑	↑	↓	↑	↑	↑
*s*_*E*_	↑	↑	↓	↑	↑	↑	↑	↑	↓	↑	↓	↓
*s*_*B*_	–	–	–	–	–	–	–	–	–	↑	↑	↑
β	↑	↑	↓	↑	↑	↓	↑	↑	↓	↑	↑	↑
τ	↑	↓	↓	↑	↓	↓	↑	↓	↑	↑	↑	↓
λ	↑	↓	↓	↑	↓	↑	↓	↑	↑	↑	↓	↓
μ	↑	↑	↓	↑	↑	↑	↑	↑	↑	↑	↑	↑

“–” in the table indicates that the parameter does not appear or is undefined in this mode.

(1) The tax differential τ reflects the tax rate gradient between the manufacturing sector and retailers. Without blockchain integration, its partial derivative sign generally satisfies $\frac{\partial w^{*}}{\partial \tau }<0,\frac{\partial P_{R}^{*}}{\partial \tau }<0,\frac{\partial \pi_{F}^{*}}{\partial \tau }<0$. The economic rationale lies in the fact that an increased tax differential incentivizes MNE to transfer profits to low-tax retail sectors by lowering transfer prices, thereby gaining tax planning benefits. However, under fair trade principles, wholesale prices to e-retailers are forced to decline synchronously, leading to lost wholesale profits. Simultaneously, lower wholesale prices stimulate online demand, intensifying channel competition and further squeezing retail profits and product prices in the MNE’s retail division, ultimately eroding total profits.

The introduction of blockchain technology triggers a critical reversal in this mechanism, particularly manifesting as $\frac{\partial w^{*}}{\partial \tau }>0$ in the composite model. This occurs because blockchain-enabled quality verification significantly enhances trust and appeal in online channels, reducing online demand’s sensitivity to wholesale prices. Consequently, MNE’s strategic focus shifts from tax avoidance to capturing trust premiums, leading them to prefer raising wholesale prices to extract greater profits directly from the online wholesale market. Nevertheless, the inherent intensified channel competition and ALP constraint effects from widening tax differentials persist. Consequently, the retail division’s product pricing remains under pressure, and total profits still decline with increasing tax differentials in most models. This indicates that technological empowerment fails to fully offset the negative channel conflict effects of tax differentials.

(2) The service level sR of the retail division and the service level *s*_*E*_ of the self-operated e-commerce platform are central to channel competition. Before blockchain adoption, enhancing retail service levels directly increased consumer utility, supported higher product pricing for MNE’s retail division, and drove overall profit growth, i.e., $\frac{\partial P_{R}^{*}}{\partial s_{R}}>0$, $\frac{\partial \pi_{F}^{*}}{\partial s_{R}}>0$. Conversely, improving self-operated e-commerce service levels boosted online channel competitiveness. While potentially raising wholesale prices slightly, it primarily diverted demand from the retail division and exerted downward price pressure, often resulting in $\frac{\partial \pi_{F}^{*}}{\partial s_{E}}<0$.

The introduction of blockchain alters this dynamic. First, regarding service levels in the retail sector, its impact reverses within the composite model. This occurs because blockchain enhances consumers’ perception of e-retailers’ service quality through parameter μ, thereby diminishing the marginal competitive advantage of retail service levels. At this point, the increased costs associated with improving retail service levels fail to yield commensurate demand premiums. Moreover, ALP may elevate internal settlement costs, ultimately eroding profits. Second, for self-operated e-commerce service levels, blockchain creates synergistic effects with the model, amplified by the influence of $\mu \beta s_{E}$. MNE can partially capture value-added by raising wholesale prices, i.e., $\frac{\partial w^{*}}{\partial s_{E}}>0$. However, the further enhancement of online competitiveness may still intensify channel conflicts under the single-channel model, leading to a decline in total profits.

(3) Blockchain technology influences MNE’s decision-making by enhancing consumer preferences for product valuation and service quality, corresponding to two core coefficients λ and μ. Partial derivative analysis reveals that both exert a positive impact on wholesale prices, i.e., $\frac{\partial w^{*}}{\partial \lambda }>0$ and $\frac{\partial w^{*}}{\partial \mu }>0$. This occurs because heightened trust directly stimulates online demand, enabling MNE to raise wholesale prices and capture premiums.

However, their transmission pathways to MNE’s total profits diverge sharply. An increase in λ primarily strengthens trust in product authenticity, intensifying the substitution effect of online channels on the retail sector and triggering fierce channel conflicts, often leading to $\frac{\partial \pi_{F}^{*}}{\partial \lambda }<0$. Conversely, an increase in μ enhances service credibility, effectively compensating for the service experience shortcomings of online channels. This promotes differentiation and complementary effects between online and offline services. This effect is particularly pronounced in composite models like “E+FBP,” where it coordinates channel relationships and enhances overall supply chain efficiency, ultimately achieving $\frac{\partial \pi_{F}^{*}}{\partial \mu }>0$. This contrast profoundly illustrates that blockchain holds greater strategic synergy value in enhancing “service process credibility” than in merely boosting “product attribute credibility.”

(4) The consumer service sensitivity coefficient β measures the weight of service in consumer decision-making. Regardless of the scenario, we have $\frac{\partial w^{*}}{\partial \beta }>0$, $\frac{\partial P_{R}^{*}}{\partial \beta }>0$, $\frac{\partial \pi_{F}^{*}}{\partial \beta }>0$. This is because an increase in β amplifies the contribution of the service component $\beta \;s_{i}$ (i = R, E, B) within the consumer utility function. This enhances the bargaining power of retail sectors with service advantages, enabling them to support higher retail and wholesale prices. Simultaneously, it incentivizes e-retailers to compete by enhancing their own services or leveraging blockchain to verify service quality. This collective effort expands the overall online market size, thereby increasing the total profitable margin of the supply chain.
(5) Consumers’ inherent preference for online channels directly influences the baseline competition between channels, corresponding to coefficients $\theta_{E}$, $\theta_{A}$, and $\theta_{B}$. Without blockchain, increased online preference typically intensifies competition for the retail sector, leading to declines in $P_{R}^{*}$ and $\pi_{F}^{*}$. With blockchain, the blockchain-enabled coefficients λ and μ interact complexly with inherent preferences. For instance, in the “E + FBP” model, blockchain may alter competitive dynamics. The increased consumer preference for self-operated e-commerce products, when empowered by blockchain, can synergize more effectively with the retail segment. This demonstrates that blockchain can mitigate channel exclusivity caused by inherent preference differences to a certain extent.

## Numerical analysis

### Analysis of the quantitative case

To enhance the support and validation of the results in this paper, an algorithmic analysis will be performed to examine the link between the impact of pertinent parameters on the total profit of the MNE, regardless of the use of blockchain technology. The baseline parameters are set as follows: *s*_*R*_=10, *s*_*E*_ = 8, *s*_*B*_ = 6, reflecting the hierarchical relationship where offline services are optimal, platform-operated services are secondary, and SOP merchants are the weakest. Consumer preferences are set as $\theta_{E}$=0.6, $\theta_{A}$=0.5, $\theta_{B}$=0.4; Tax differential τ=0.2, service sensitivity coefficient β= 0.8; blockchain empowerment coefficients λ= 1.25, μ= 1.67, satisfying constraint (3); blockchain introduction cost *F* = 10; platform commission rate x = 0.1, franchise fee f = 1, unit product service fee c = 0.05. Model parameter values primarily reference typical settings and relative relationships from relevant literature [13,49], aiming to construct a representative scenario with sufficient differentiation to ensure the rationality of its economic implications.

#### The influence of tax disparities on the overall profit of the MNE from a blockchain perspective.

**Fig 7:**
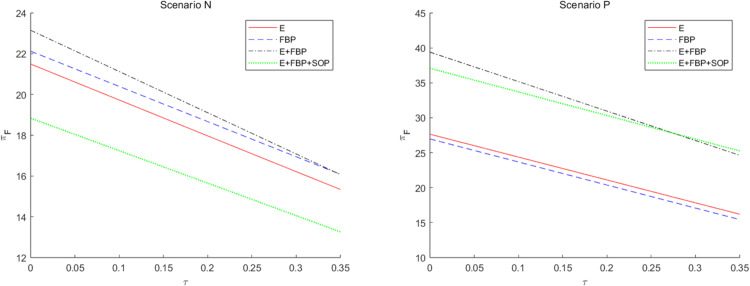
The impact of tax disparities on the overall profit of the MNE employing different entry models.

Fig 7 presents an analysis of the tax disparities on the overall earnings of the MNE across the four e-commerce entry models. This numerical research indicates that the total profit of the MNE diminishes with increased tax disparity, irrespective of the implementation of blockchain technology.

According to the results of the comparative static analysis, $\frac{\partial \pi_{F}^{*}}{\partial \tau }<0$, indicating that tax differentials exert a consistent negative marginal effect on the total profits of MNE. The underlying mechanism is that while a widening tax gap theoretically incentivizes MNE to shift profits to low-tax jurisdictions by lowering wholesale prices for tax planning gains, the existence of the ALP rule forces them to simultaneously reduce wholesale prices to e-retailers. This creates an opportunity for e-retailers to act as free riders, resulting in a partial loss of wholesale profits. As the tax gap increases, the E-retail market expands, and competition in the downstream sector intensifies, it becomes challenging for MNE’s manufacturing divisions to achieve higher anticipated profits from product sales while the substantial tax burden further diminishes their retail profits.

Furthermore, it is evident that when the MNE introduced blockchain technology, it gained more significant advantages in the marketplace model of the composite e-commerce entry framework of “E+FBP” compared to “E+FBP+SOP.” The MNE implementing blockchain technology may enhance the reputation of e-retailers, hence augmenting market potential; consequently, the adoption of blockchain by the MNE serves as a quality assurance for e-retailers. Consequently, customers will enhance their trust and preference for the e-retailer’s offerings, the e-retailer will augment the order volume of the MNE’s items, and the MNE will realize increased wholesale profits. This manifests as the introduction of blockchain-enabled parameters λ and μ, which raise the demand function intercept for online channels, thereby increasing the optimal wholesale price at equilibrium. As shown in Table 5, $\frac{\partial w^{*}}{\partial \lambda }>0$, and $\frac{\partial w^{*}}{\partial \mu }>0$. Nevertheless, the initial market potential for e-retailers is substantial within the monotypic e-commerce competition framework, exemplified by “E only” and “FBP only.”

Furthermore, implementing blockchain technology by MNEs has a negligible impact on enhancing consumer preference for e-retailers. Consequently, the expansion of wholesale profit remains constrained, indicating that the adoption of blockchain technology does not yield a notable increase in profit margins. In the composite e-commerce entry model, such as “E+ FBP” and “E+FBP+SOP” models, the e-commerce platform engages with FBP merchants through the services offered, while the presence of SOP merchants diminishes consumers’ expectations of e-retailers overall. The initial market potential of composite e-retailers is thus limited, whereas the introduction of blockchain technology has significantly enhanced customer confidence in e-retailer items, resulting in a rise in MNE orders. Despite diminished tax planning advantages resulting from tax differences, wherein a substantial portion of earnings is maintained inside the manufacturing division of the MNE in high-tax jurisdictions, the augmentation of wholesale profits mitigates this adverse impact.

#### The influence of services provided by the e-commerce platform, services provided by the retail division of the MNE on the overall profit of the MNE from a blockchain perspective.

**Fig 8:**
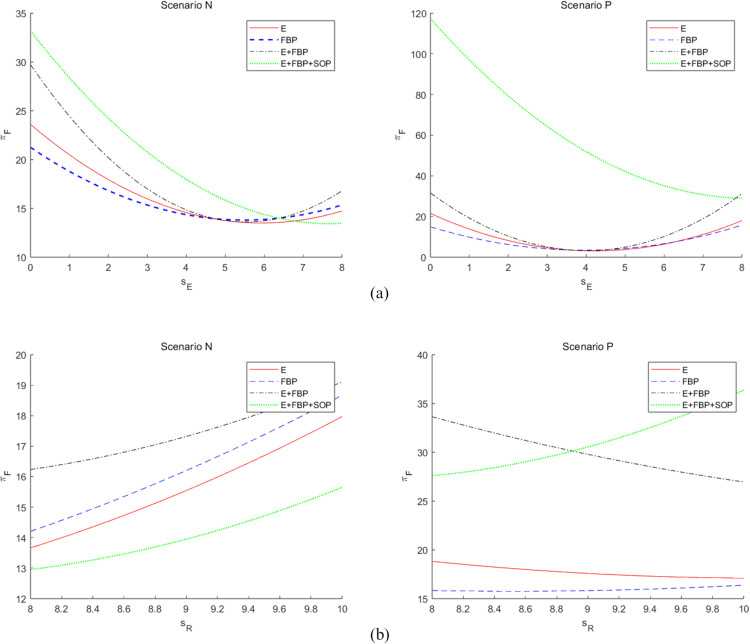
The impact of the service provided by the e-commerce platform and the service offered by the retail division of the MNE on the overall profits of the MNE employing different entry models. The impact of (a) the service level provided by self-operated e-commerce platforms on the total profit of the MNE choosing different e-commerce entry models, and (b) the impact of the service level provided by the retail sector on the total profit of the MNE choosing different e-commerce entry models, with and without blockchain technology introduction.

Fig 8 presents an analysis of the service provided by the e-commerce platform and the service offered by the retail division of the MNE on the overall profits of the MNE across the four e-commerce entry models.

Fig 8 (a) illustrates an algorithmic examination of the overall profit of the MNE derived from the services offered by the self-operated e-commerce division across the four e-retailer entry models. This numerical research indicates that the MNE’s overall profit diminishes as the services offered by Self-operated e-commerce increase, irrespective of the MNE’s decision to implement blockchain technology. This “bottoming-out rebound” phenomenon reflects the nonlinear characteristic of the profit function $\pi_{F}^{*}$, which first decreases and then increases with changes in *s*_*E*_. The underlying mathematical mechanism is that profit changes are influenced by two opposing effects. On one hand, increasing *s*_*E*_ intensifies channel competition, reducing retail sector profits; on the other hand, increasing *s*_*E*_ also expands the online market size, boosting wholesale profits. When *s*_*E*_ is low, the negative effect dominates, causing total profits to decline. As *s*_*E*_ continues to increase, the positive effect gradually strengthens. At a critical point $s_{E}^{0}$, the two effects balance precisely, leading profits to reach a local minimum. Beyond $s_{E}^{0}$, the positive effect begins to dominate, causing total profits to rise. Numerical simulations confirm that under given parameters, the critical point $s_{E}^{0}\approx 7.5$ is consistent with theoretical analysis.

The superior service level offered by self-operated e-commerce would mitigate the service shortcomings of the MNE’s manufacturing division compared to the self-operated division with FBP merchants, enhancing customer preference for e-retailers’ items. The rise in sales volume leads to an increase in product orders from e-retailers to the MNE, prompting the MNE to elevate the wholesale price. Consequently, substantial profits remain within the manufacturing division of the MNE located in high-tax regions, diminishing the advantages of tax planning. Simultaneously, in accordance with the ALP regulation, the MNE’s transfer price for the manufacturing division inside the retail division escalates, adversely impacting retail earnings. Nevertheless, when the service quality of the captive platform approaches that of the MNE’s manufacturing division, the substantial wholesale profit from the e-retailer’s numerous orders will counterbalance the effects of diminished tax planning benefits and retail profit. Consequently, when the services offered by self-operated e-commerce attain a specific threshold, the overall profitability of MNE tends to stabilize and then increase.

Fig 8 (b) illustrates a numerical examination of the influence of the services rendered by the MNE’s manufacturing division on the overall profit of the MNE across the four e-retailer entry models. This numerical analysisindicates that, in the absence of blockchain technology, the MNE’s total profit rises according to the level of services offered by its manufacturing division. Conversely, when blockchain technology is implemented, the total profit similarly increases with the level of services from the manufacturing division, except for the e-commerce entry model of “E+FBP+SOP.” This significant finding aligns with the comparative static analysis results presented in Tables 4 and 5. Without blockchain introduced, $\frac{\partial \pi_{F}^{*}}{\partial s_{R}}>0$; with blockchain introduced, under the “E + FBP” model, $\frac{\partial \pi_{F}^{*}}{\partial s_{R}}<0$. The mathematical reason for this sign reversal lies in the altered mechanism of *s*_*R*_ within the profit function after introducing the blockchain parameters λ and μ. Solving the equilibrium solution of Proposition 3 and calculating $\frac{\partial \pi_{F}^{*}}{\partial s_{R}}$ reveals that this derivative contains terms (λ-1) and (μ-1). When λ>1 and μ > 1, these terms alter the sign of the derivative. With the introduction of blockchain technology, the MNE’s overall profit diminishes as the service level offered by the manufacturing division escalates. An enhancement in the service quality offered by the MNE will certainly elevate consumer preference for its manufacturing division’s products, particularly luxury goods. Consequently, the retail division of the MNE will be further favored by consumers who are typically resistant to persuasion. Nevertheless, when the MNE implements blockchain technology, the increasingly enhanced service levels in its manufacturing division will diminish the beneficial effects of blockchain for e-retailers, resulting in reduced wholesale margins, elevated retail margins, and diminished tax planning advantages that will fail to offset the expenses associated with the adoption of blockchain technology. The “E+FBP+SOP” e-commerce entry model, due to its limited initial market potential, will enhance consumer trust in e-retailers’ products following the implementation of blockchain technology by MNE, resulting in increased wholesale profits that will offset the costs associated with blockchain integration.

In Fig 8 (a) and (b), it is evident that when the MNE implements blockchain technology, the total profit of the MNE markedly increases in the composite e-commerce entry models, namely “E+FBP” and “E+FBP+SOP.” The initial market potential of the composite e-commerce entry model is minimal; nevertheless, the integration of blockchain technology significantly enhances customer desire for e-retailers’ items, resulting in increased wholesale profits. Simultaneously, due to the ALP regulation, the MNE experienced more significant overall gains, despite a reduction in its retail earnings.

#### The influence of consumers’ service sensitivity coefficients on the overall profit of the MNE from a blockchain perspective.

**Fig 9:**
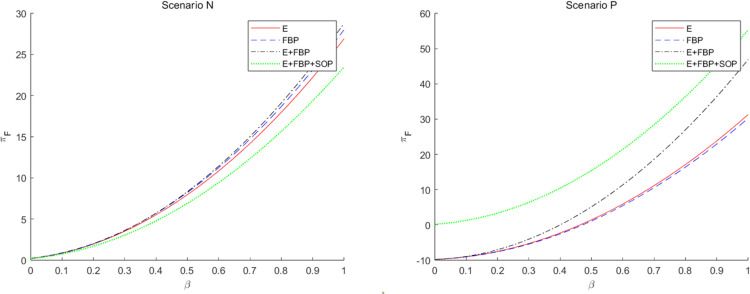
The influence of customers’ service sensitivity on the overall profit of the MNE employing different entry models.

Fig 9 numerically illustrates the effect of the customer service sensitivity factor on the MNE’s overall profit across the four e-retailer entry strategies. This numerical analysis indicates that without introducing blockchain technology, the MNE excels in monotypic e-commerce entry models such as “E only” and “FBP only.” Conversely, with the implementation of blockchain technology, the MNE demonstrates superior performance in the “E+FBP” and “E+FBP+SOP” entry models.

This phenomenon can be explained by comparing the magnitude of $\frac{\partial \pi_{F}^{*}}{\partial \beta }$ across different modes. According to Tables 4 and 5, $\frac{\partial \pi_{F}^{*}}{\partial \beta }>0$ in all models, though its numerical magnitude varies by mode. By calculating the coefficients of the partial derivatives of the profit function with respect to β across modes, we observe: without blockchain introduction, the coefficient is larger in the single-type mode; after blockchain introduction, the coefficient is larger in the composite-type mode. This aligns with the observed shift in model advantages discussed earlier. Initially, when the online marketplace operates under a monotypic e-retailer entry model, consumers’ heightened significance to service enhances the overall service quality. Superior service can also expand pricing flexibility and elevate product demand, subsequently augmenting the volume of orders for MNE’s products on the online platform. In a composite e-commerce entry model, e-commerce platforms would affect customer demand for FBP merchants’ items through services to maintain the competitiveness of self-operated e-commerce products. Decreased service levels in the online marketplace will result in diminished customer preference for e-retailer items, leading to a decline in sales of e-retailer products and a reduction in the number of orders from the MNE. The manufacturing component of the MNE cannot restore its impetus at this juncture. Secondly, the MNE’s decision to implement blockchain technology will heighten customer sensitivity to service quality. While the MNE’s manufacturing division consistently delivers superior service quality, the MNE will achieve more profitability through a composite e-commerce entry model when customer service sensitivity is elevated.

Simultaneously, when the MNE implements blockchain technology, it incurs earnings losses when consumer service sensitivity is low. Implementing blockchain technology by the MNE will elevate consumer expectations regarding e-retailers’ products, resulting in enhanced trust and preference for these products. Consequently, this will lead to market encroachment within the retail division of the MNE and diminished retail profits for them. Simultaneously, while implementing blockchain technology by the MNE enhances the service quality of the e-retailer’s online platform, customer sensitivity to service is now minimal. The marginal enhancement in the service level of the e-retailer has a negligible impact on augmenting customer utility and demand for the e-retailer’s items, resulting in a slight rise in the number of orders for the MNE’s products. The wholesale profit from the items has minimal impact and is inadequate to counterbalance the losses incurred by the retail division of the MNE and the implementation of blockchain technology. Regardless of the e-commerce entry model chosen by the MNE, achieving standard returns is unattainable, and it is imprudent to implement blockchain technology.

#### The influence of the MNE’s involvement in blockchain on customer preferences for product value and service on the overall profit of the MNE from a blockchain perspective.

**Fig 10:**
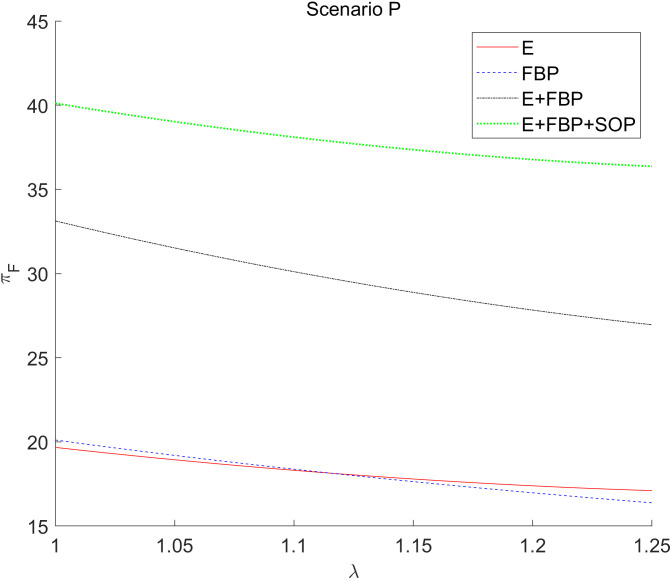
The effect of the sensitivity coefficient λ of consumers’ preference for product value to the introduction of blockchain technology by the MNE on the total profits of the MNE.

Fig 10 illustrates that the overall profit of the MNE diminishes when the sensitivity coefficient of customers’ demand for product value escalates.

The increasing initial market potential of e-retailers leads to a subsequent decline in the utility of blockchain technology, a reduction in wholesale profit growth margins, and concurrently, an adverse effect on the retail profits of the manufacturing division of the MNE. This observation aligns with the results of the comparative static analysis in Table 4, namely that under all e-commerce entry models, $\frac{\partial \pi_{F}^{*}}{\partial \lambda }<0$.

**Fig 11:**
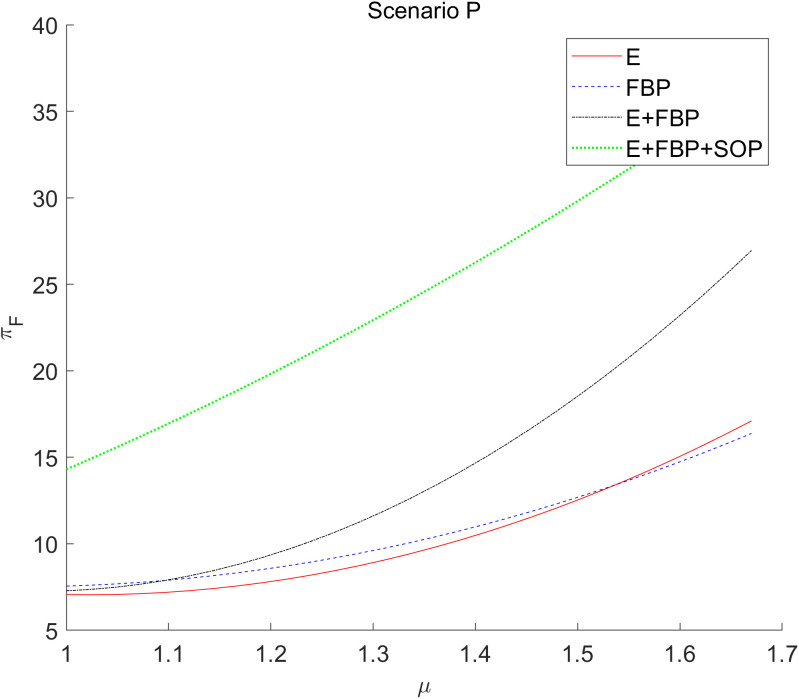
The effect of the sensitivity coefficient of consumers’ preference for service to the introduction of blockchain technology by the MNE on the total profits of MNE.

Fig 11 illustrates that the overall profit of the MNE rises when the sensitivity coefficient of customer demand for product services grows.

The elevated sensitivity coefficient of customer preference for product services signifies that the enhancement in consumer preference for e-retailers due to blockchain technology would be more pronounced, resulting in substantial wholesale profits for the MNE. In the context of service, the manufacturing division of the MNE possesses distinct benefits, including expertise and infrastructure, which will become increasingly indispensable, particularly for luxury products. Consequently, even if customers exhibit a heightened preference for e-retailers, the manufacturing division of the MNE will not experience significant losses.

### Analysis of the decision domain

This section will employ a quantitative research methodology to examine the interactions between the service level provided by self-operated e-commerce, the service level provided by the MNE’s retail division, and service sensitivity coefficients. The objective is to analyze how the MNE should select the e-commerce entry model for optimal decision-making without blockchain technology and following its implementation.

#### Analysis of service levels between self-operated e-commerce versus the manufacturing section of the MNE.

**Fig 12:**
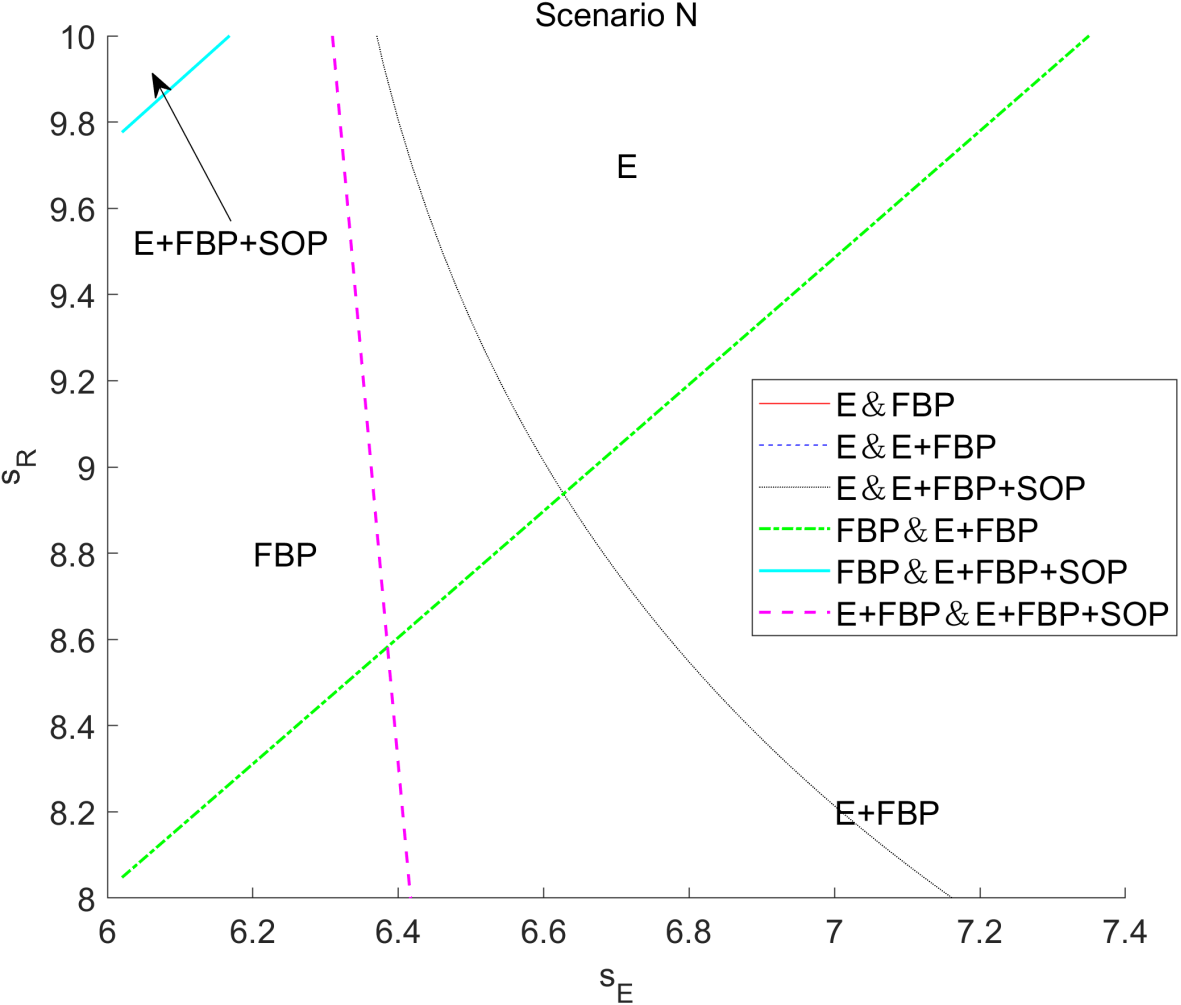
The impact of the service level provided by self-operated e-commerce versus the service level provided by MNE’s manufacturing division on the choice of the e-commerce entry model without the introduction of blockchain technology.

Fig 12 illustrates that the impact of the service level provided by self-operated e-commerce versus the service level provided by MNE’s manufacturing division on the choice of the e-commerce entry model without the introduction of blockchain technology.

When the service level of self-operated e-commerce is between 6 and 6.2 and the service level of the MNE’s manufacturing division is between 9.8 and 10, the MNE will opt for the “E+FBP+SOP” entry model. During this period, there is a significant difference in service levels between e-retailers and the MNE. The manufacturing division of the MNE enjoys a marked competitive edge, and its retail profits can compensate for the decline in wholesale profits. When the service level of self-operated e-commerce merchants is from 6 to 6.65, and the service level of the MNE’s manufacturing division is between 8 and 10, the MNE will choose the “FBP only” entry model. Conversely, when the service level of self-operated e-commerce merchants is from 6.65 to 7.4, and the service level of the MNE’s manufacturing division is from 8.9 to 10, the MNE will select the “E only” entry model. It is attributable to the fact that as the service gap between e-retailers and the manufacturing division of the MNE widens, only a monotypic e-commerce entry model with higher consumer preference can maintain elevated wholesale margins. In the monotypic e-commerce entry model, consumer preference for FBP merchant products is relatively lower. Hence, when the service level of the self-operated e-commerce entry model falls below a certain threshold, the MNE will opt for the e-commerce entry model with only FBP merchants to sustain a higher retail profit. FBP merchants deliver services to consumers via self-operated platforms. As FBP merchants’ products gain increasing consumer favor, platforms will progressively lower service levels to cut costs. In contrast, for the self-operated e-commerce entry model only, self-operated platforms are willing to provide high-quality services to secure a broader pricing space, boosting revenue. Therefore, when the service level provided by the self-operated platform reaches a certain level, the MNE should choose the “E only” entry model to enhance wholesale profit. When the service level of self-operated e-commerce is from 6 to 7.4, and the service level of the MNE’s manufacturing division is between 8 and 10, the MNE will choose the “E+FBP” e-commerce entry model. As the service gap between the e-retailer and the manufacturing division of the MNE narrows, the MNE’s retail profit is jeopardized. Only when FBP merchants and self-operated e-commerce compete with e-retailers will the e-commerce platform consider whether their services impact their products. They may then employ pricing strategies to increase revenue, which correspondingly raises the MNE’s wholesale margins.

**Fig 13:**
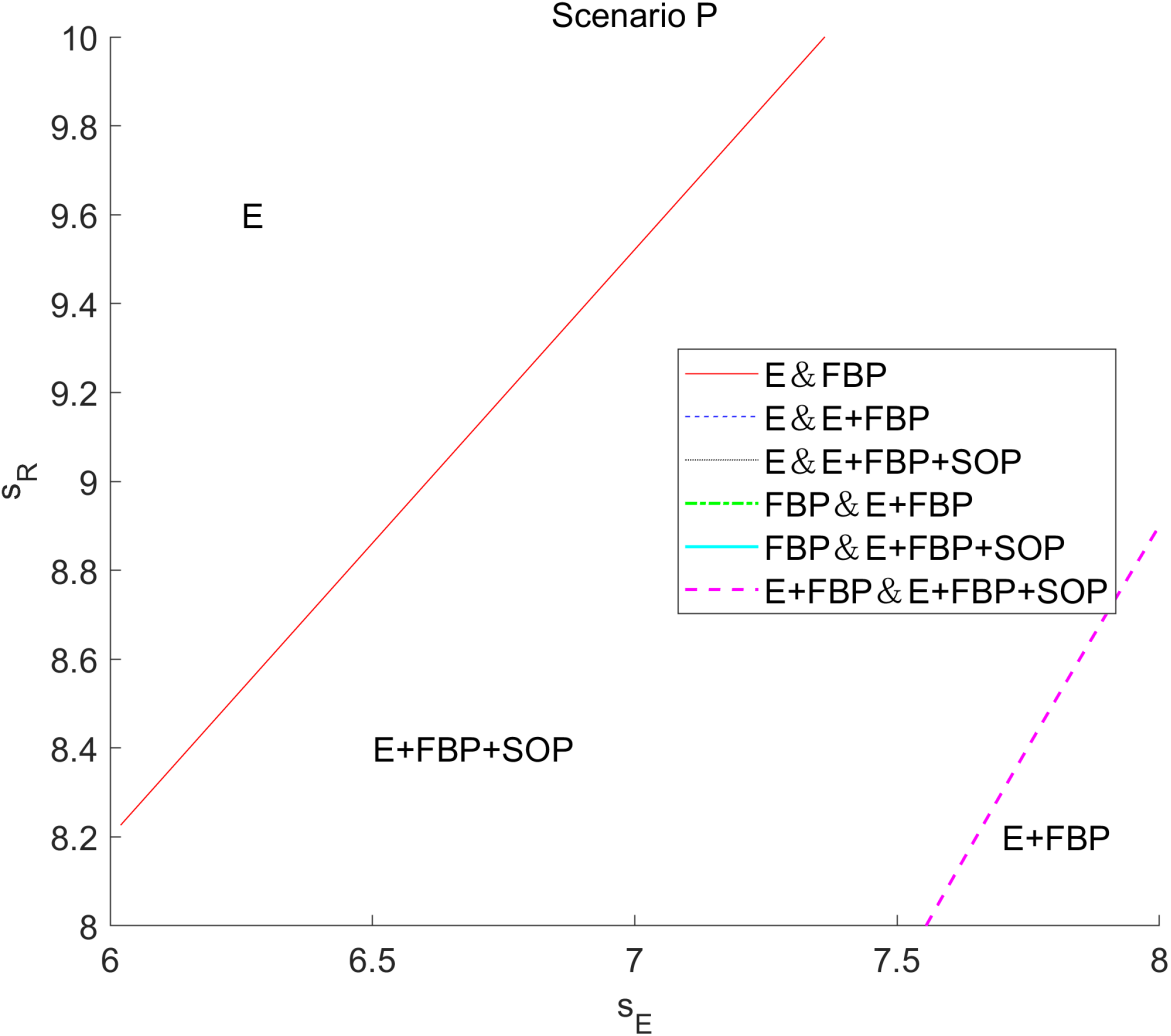
The impact of the service level provided by self-operated e-commerce versus the service level provided by MNE’s manufacturing division on the choice of the e-commerce entry model with the introduction of blockchain technology.

Fig 13 illustrates that the impact of the service level provided by self-operated e-commerce versus the service level provided by MNE’s manufacturing division on the choice of the e-commerce entry model with the introduction of blockchain technology.

When the service level of self-operated e-commerce is from 6 to 7.5, and the service level of the manufacturing division of the MNE is between 8.2 and 10, in the region above the red line, the MNE will choose the “E only” e-commerce entry model. A substantial service disparity exists between e-retailers and the manufacturing division of the MNE, while the retail division possesses a considerable competitive edge. The “E only” e-commerce entry model possesses significant initial market potential; however, implementing blockchain technology will exert minimal influence on e-operated e-commerce. Consequently, the MNE may sustain a specific degree of wholesale profitability while achieving more significant retail margins. In the region beneath the red line, MNE will select the “E+FBP+SOP” e-commerce entry model. A small service gap exists between e-retailers and the manufacturing division of the MNE. However, the existence of SOP merchants undermines customer trust in e-retailers, therefore allowing the retail division of the MNE to maintain a considerable competitive advantage. Simultaneously, the “E+FBP+SOP” e-commerce entry model, characterized by a low initial market potential, significantly enhances consumer preference for e-retailers by implementing blockchain technology. This advancement motivates the MNE to escalate wholesale prices to secure greater profits. Despite the limitations imposed by the ALP regulation and the detrimental impact on the retail division’s profits, the wholesale profits are adequate to counterbalance this adverse effect. When the service level of self-operated e-commerce ranges from 7.5 to 8, and the service level of the MNE’s manufacturing division ranges from 8 to 8.85, the MNE will choose the “E+FBP” entry model. The competitive advantage of the MNE retail division is minimal; however, the “E+FBP” e-commerce entry model presents a moderate initial market potential. The implementation of blockchain technology significantly impacts e-retailers. Due to the trade-offs and competition between self-operated e-commerce merchants and FBP merchants, the MNE will sustain its retail manufacturing division while experiencing an increase in wholesale profits. As the service gap diminishes and e-retailers become increasingly competitive, the MNE benefits more from an e-commerce entry strategy when the e-retailer possesses moderate initial market potential.

When comparing the optimal decision-making alterations of the MNE with and without the introduction of blockchain technology, it is evident that if the service level offered by self-operated e-commerce ranges from 6 to 7.5, and the service level provided by the MNE’s manufacturing division is between 8.2 and 10, the MNE will select three e-commerce entry models based on varying circumstances without blockchain. However, with the introduction of blockchain technology, the MNE will exclusively opt for the “E only” entry model. The MNE’s retail division is less challenged by the e-retailer when the disparity between it and the services offered by self-operated e-commerce is significant, resulting in considerable retail margins and little wholesale margins. The “E+FBP+SOP” composite e-commerce entry model exhibits limited initial market potential; nevertheless, the use of blockchain technology by the MNE will significantly enhance the e-retailer’s credibility, hence augmenting order volume and, consequently, the wholesale profit of the MNE. The current profit growth of the MNE mainly derives from wholesale profits. However, due to tax disparities, this growth is concentrated in the high-tax manufacturing division, diminishing the MNE’s tax coordination benefits.

#### Analysis of consumers’ service sensitivity factor versus the level of service provided by Self-operated e-commerce.

**Fig 14:**
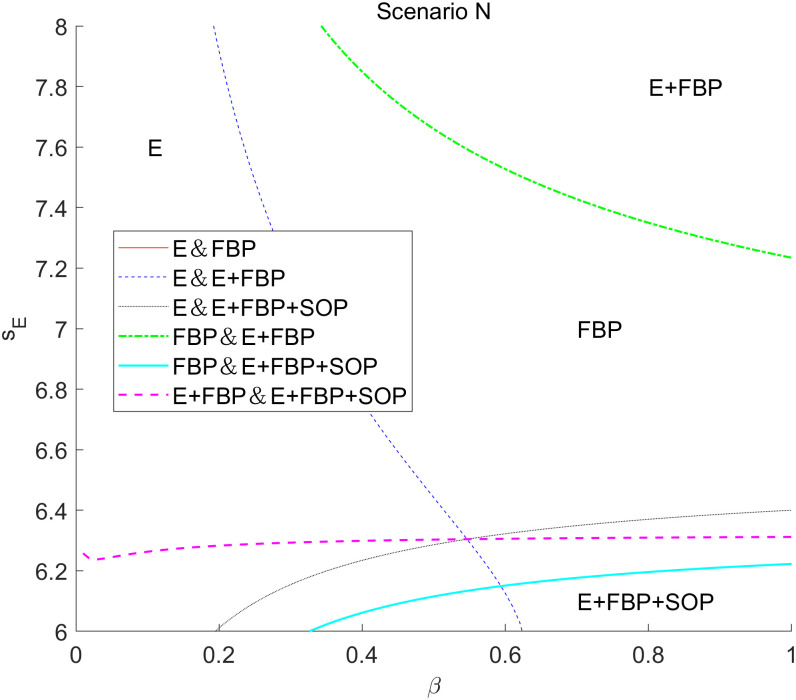
The impact of consumers’ service sensitivity factor versus the level of service provided by Self-operated e-commerce on the choice of the e-commerce entry model without the introduction of blockchain technology.

Fig 14 illustrates that the impact of consumers’ service sensitivity factor versus the level of service provided by Self-operated e-commerce on the choice of the e-commerce entry model without the introduction of blockchain technology.

When the customer service sensitivity coefficient ranges from 0 to 0.55, and the service level offered by Self-operated e-commerce is between 6 and 8, the MNE will choose the “E only” e-commerce entry strategy. When consumers exhibit a low service sensitivity coefficient, the elevated service level of Self-operated e-commerce diminishes the service disparity with the manufacturing division of the MNE, enabling the MNE to sustain a higher wholesale profit. Conversely, when consumers demonstrate a high service sensitivity coefficient, the reduced service level of Self-operated e-commerce can lower service costs, prompting a price adjustment of the product to attract consumers, thereby increasing the MNE’s retail profit. When the customer service sensitivity coefficient ranges from 0.35 to 1, and the service level offered by Self-operated e-commerce is between 7.2 and 8, the MNE will choose the e-commerce entry model of “E+FBP.” Currently, consumers prioritize the usefulness of service quality. When self-operated e-commerce delivers superior service, consumers get more excellent utility from the products of both self-operated e-commerce and FBP merchants, resulting in increased wholesale profits for the MNE. Despite a portion of retail earnings being forfeited at this juncture, the advantages of tax planning compensate for part of the deficit. When the customer service sensitivity coefficient ranges from 0.2 to 1, and the service level offered by Self-operated e-commerce is between 6.2 and 8, the MNE will choose the “FBP only” entry model. Consumers increasingly prioritize service quality; thus, when the service level of self-operated e-commerce is moderate, the MNE will opt for the “FBP only” e-commerce entry model to maximize retail profits while sustaining a reasonable wholesale profit. When the customer service sensitivity coefficient is from 0.35 to 1, and the service level offered by Self-operated e-commerce is between 6 and 6.2, the MNE will choose the “E+FBP+SOP” e-commerce entry model. Consumers exhibit heightened sensitivity to service quality; hence, the worse service levels of self-operated e-commerce and the inclusion of SOP merchants diminish the overall value of the e-retailer’s items. Consequently, the manufacturing division of MNE possesses a substantial competitive advantage. Consequently, the MNE will relinquish wholesale revenues and concentrate on securing greater retail profits and tax optimization advantages.

**Fig 15:**
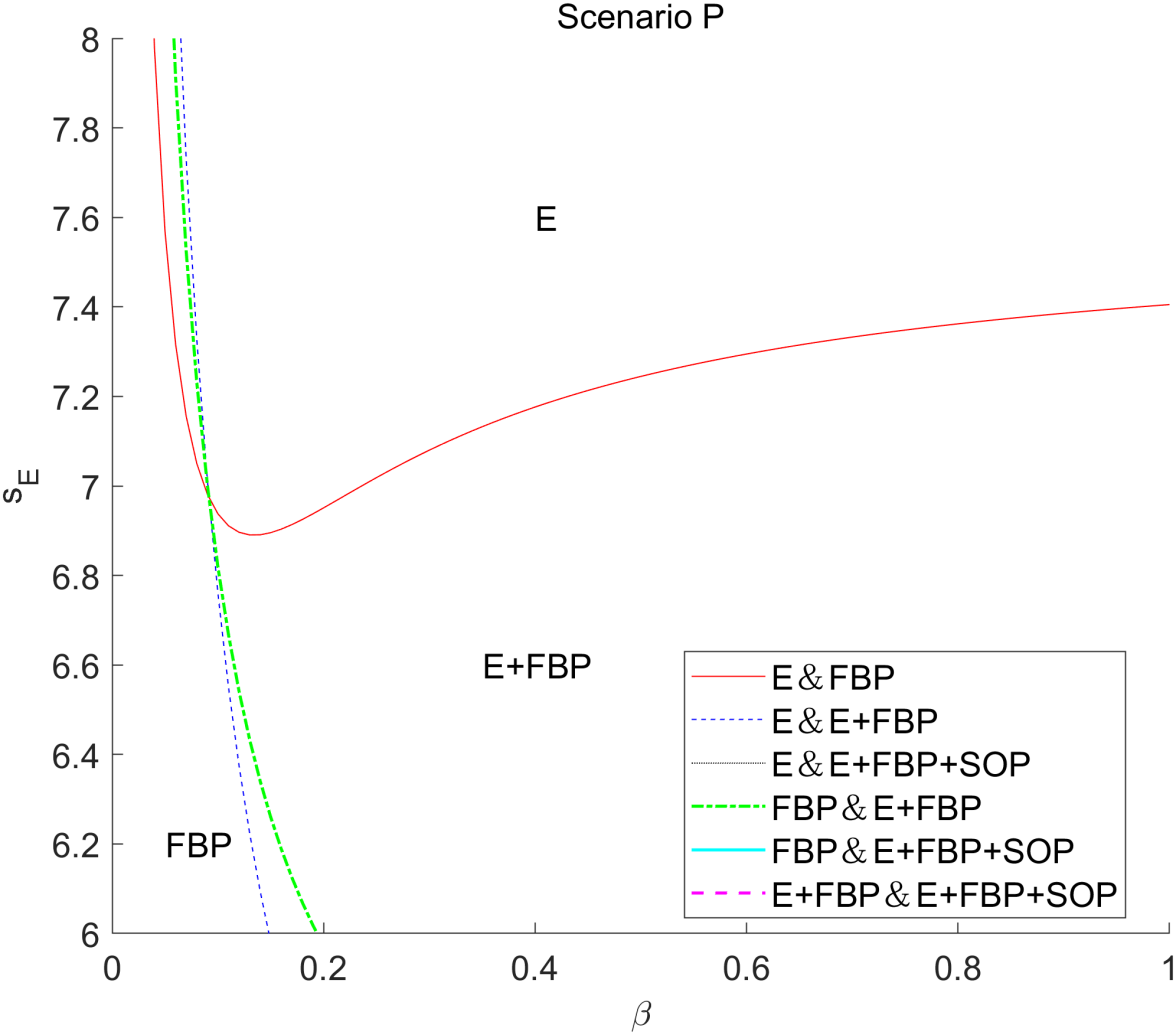
The impact of consumers’ service sensitivity factor versus the level of service provided by Self-operated e-commerce on the choice of the e-commerce entry model with the introduction of blockchain technology.

Fig 15 illustrates that the impact of consumers’ service sensitivity factor versus the level of service provided by Self-operated e-commerce on the choice of the e-commerce entry model with the introduction of blockchain technology.

When the customer service sensitivity coefficient is from 0 to 0.2, and the service level offered by self-operated e-commerce merchants is between 6 and 8, the MNE will choose the “FBP only” entry model. Currently, customers exhibit insensitivity to the utility improvements provided by services, so when the MNE introduces blockchain technology, the enhanced service quality of e-retailers will provide minimal utility benefits for consumers. If e-retailers exclusively engage FBP merchants, the e-commerce market’s initial potential remains substantial; thus, implementing blockchain technology will not significantly enhance consumer product utility in e-retailers, negatively impacting the manufacturing division of the MNE. Consequently, selecting the e-commerce entry model of “FBP only” will enable the MNE to enhance their wholesale margins and stabilize their retail margins. When the customer service sensitivity coefficient ranges from 0.1 to 1, and the service level offered by self-operated e-commerce merchants is between 6.9 and 8, the MNE will choose the “E only” e-commerce entry model. In this scenario, consumers highlight the increased utility resulting from enhanced service quality, with self-operated e-commerce offering superior service levels. Consequently, when the MNE implements blockchain technology, the improved service quality from e-retailers will significantly augment consumer utility. When only self-operated e-commerce exists among e-retailers, elevated consumer preferences and service standards will drive self-operated e-commerce to raise product prices, boost wholesale order volumes, and shift service costs to consumers, thereby maximizing profits. Consequently, the MNE will choose the “E only” entry model, which will significantly enhance wholesale profits, albeit potentially diminishing some retail profits and tax planning advantages. When the customer service sensitivity coefficient ranges from 0.15 to 1, and the service level offered by self-operated e-commerce is between 6 and 7.4, the MNE will choose the “E+FBP” entry model. Currently, consumers prioritize enhancing service quality resulting from increased utility; nevertheless, the e-commerce platform offers a diminished level of service. The initial market potential of the “E+FBP” e-commerce entry model is moderate; hence, following the implementation of blockchain technology by the MNE, customer trust in e-retailers’ products will be enhanced, while the manufacturing division will retain a significant advantage. Simultaneously, when just FBP merchant items compete with the platform’s exclusive offerings, e-retailers will have comparatively advantageous selling prices owing to reduced service expenses, attracting certain consumer purchasing behaviors. At the same time, the MNE would also realize increased wholesale profits. Consequently, the MNE elects the “E+FBP” self-operated e-commerce entry strategy that may preserve retail profits while achieving elevated wholesale margins.

When comparing the optimal decision changes of the MNE with and without blockchain technology, it is observed that if the consumer service sensitivity coefficient ranges from 0 to 0.2, and the service level offered by self-operated e-commerce merchants is between 6 and 8, the MNE will opt for the “E only” e-commerce entry model without blockchain technology. In contrast, it will select the “FBP only” entry model upon introducing blockchain technology. The primary reason for this is that consumer sensitivity to service is currently low, and the benefits of service improvement are not apparent to consumers; therefore, for the MNE to achieve overall profit enhancement, it must primarily concentrate on increasing retail profits. The FBP merchants offer a comparable service level but have a lower consumer preference than self-operated e-commerce merchants. Consequently, the MNE will opt for the “FBP only” entry model to ensure the stability of wholesale profits while simultaneously enhancing retail profits. When the service sensitivity coefficient of consumers ranges from 0.1 to 1, and the service level offered by self-operated e-commerce is between 6.9 and 8, without blockchain integration, the MNE will opt for the “E+FBP” entry model when the service level is elevated, and the “FBP only” e-commerce entry model when the service level is diminished. However, with blockchain implementation, the MNE will select the “E only” entry model. At this juncture, both consumer sensitivity to services and the offerings of e-commerce platforms are elevated. The service disparity between self-operated e-commerce and FBP merchants, as well as the retail division of the MNE, is minimal, preventing the retail division from securing consumer preference through an absolute advantage. Consequently, when the MNE implemented blockchain technology, consumer confidence in e-retailers with lower initial market potential increased significantly, while confidence in e-retailers with higher initial market potential saw a lesser increase. If the entrance model of e-retailers is not adjusted, the MNE will forfeit retail profits owing to significant shifts in customer preferences. It is evident that while the MNE may generate increased wholesale profits, the overall profit would remain diminished due to tax disparity and reduced tax planning advantages. When the consumer service sensitivity coefficient ranges from 0.35 to 1, and the service level offered by self-commerce e-commerce is between 6 and 6.2, the MNE will opt for the “E+FBP+SOP” entry model without the introduction of blockchain technology but will select the “E+FBP” e-commerce entry model following the implementation of blockchain technology. Currently, consumer sensitivity to service is heightened, while the service quality offered by e-commerce platforms is diminished. Consequently, the disparity in service between self-operated e-commerce and FBP merchants, as well as the retail divisions of the MNE, is more pronounced, leading consumers to prefer purchasing from manufacturing divisions that provide a superior service experience. Therefore, once the MNE has adopted blockchain technology, failure to adjust their e-retailer entry model will lead to retail profit losses triggered by pronounced shifts in consumer preferences.

## Conclusion

### Research findings

The paper categorizes online sales channels in the cross-border e-commerce sector into Self-operated e-commerce, FBP merchants, and SOP merchants. In this paper, we develop a multinational dual-channel supply chain model that comprises an MNE’s manufacturing and retail divisions, alongside e-retailers, on a cross-border e-commerce platform. It examines how varying entry model selections by e-retailers influence the overall economic efficiency of the MNE based on an analysis of consumer utility preferences and product demand. We employ Stackelberg’ s game theory to determine the optimal retail price for each division, the optimal wholesale price, and the optimal total profit function for the MNE across four e-tailer entry modes,i.e., “E only,” “FBP only,” “E+FBP,” and “E + FBP + SOP.”

We initially formulate the utility preference function of consumers and derive the demand function for products across various retail divisions. It subsequently establishes the profit function for each division of interest, predicated on the assumption that consumer demand for products corresponds to the quantity purchased and that the quantity sold in each retail division aligns with the wholesale quantity in the manufacturing division. We examines the effects of tax disparities, services rendered by self-operated e-commerce compared to those offered by the manufacturing division of the MNE, the service sensitivity coefficient, and the sensitivity coefficient of consumer preference for product value due to MNE involvement in blockchain, alongside the sensitivity coefficient of consumer preference for product services resulting from MNE involvement in blockchain, on the overall profits of the MNE, through the resolution of the equilibrium strategy and numerical analysis. We explore the impact of the service level of Self-operated e-commerce, the service level of the MNE’s retail division, and the consumer service sensitivity coefficient on the decision-making process regarding the e-retailer entry model in the context of the MNE’s online channel, with the perspective of whether the MNE introduce blockchain technology. This research offers a significant theoretical framework for optimizing the selection strategy of the MNE’s e-commerce entry model. Through theoretical examination and numerical fitting, the principal results of this article are as follows:

(1) The larger the tax disparity, the lower the overall profits of MNEs, irrespective of the implementation of blockchain technology. The overall profit of the MNE diminishes with the escalation of tax disparity, irrespective of the introduction of blockchain technology. More considerable tax disparities often compel MNEs to reduce wholesale prices to keep greater profits inside manufacturing divisions in low-tax jurisdictions and capitalize on tax planning advantages. Nevertheless, the ALP regulation mandates that the MNE align the decrease of wholesale prices for items sold to e-retailers, resulting in a loss of certain wholesale earnings. As the tax gap increases, the E-retail market expands, and rivalry in the downstream sector intensifies, it becomes challenging for the manufacturing division of the MNE to achieve its anticipated profits from product sales. The substantial tax burden will further endanger the MNE’s retail profitability. This discovery aligns with that of Baozhuang Niu (2021) [21]. The services offered by Self-operated e-commerce, the manufacturing division of MNE, the sensitivity coefficient of consumer services, and the sensitivity coefficient of consumer preference for product value due to MNE’s involvement in blockchain demonstrate a nonlinear relationship with MNE’s total profit. It has been observed that irrespective of the MNE’s decision to implement blockchain technology, the overall profit diminishes as the service level of Self-operated e-commerce escalates, while it grows with a rise in the service sensitivity coefficient. Without the introduction of blockchain technology, the total profits of the MNE diminish as the level of service from self-operated e-commerce rises and increases as the retail division’s service level rises. Conversely, with the introduction of blockchain technology, total profits decline with an increase in the retail division’s service level, rise with an increase in the service sensitivity coefficient, decrease as the consumer preference sensitivity coefficient for product value escalates, and increase as the consumer preference sensitivity coefficient for product service rises.
(2) The analysis reveals that the MNE will achieve a more significant total profit by selecting a singular e-commerce entry model without blockchain technology. In contrast, when blockchain technology is implemented, they will attain higher total profit by opting for a composite e-commerce entry model. The implementation of blockchain technology by the MNE serves as a quality assurance mechanism for e-retailers. Consequently, customers will enhance their trust and preference for the e-retailer’s offerings, prompting the e-retailer to augment orders for the MNE’s items, enabling the MNE to achieve more wholesale profits. The initial market potential of e-commerce entry models like “E only” and “FBP only” is substantial, yet the incorporation of blockchain technology does not yield a notable profit increase for the MNE. Conversely, composite e-commerce entry models such as “E+FBP” and “E+FBP+SOP” exhibit low initial market potential; however, the implementation of blockchain technology significantly enhances consumer trust in e-retailers’ products, resulting in a considerable boost in wholesale profitability for the MNE.
(3) When the MNE introduces blockchain technology, a small service gap between the e-retailer and the MNE’s manufacturing division suggests the MNE should adopt an e-commerce entry model with moderate initial market potential for the e-retailer. When the service level of the retail division of the MNE is constant, an increase in the service level of the self-operated e-commerce division results in a diminishing service gap between e-retailers and the MNE’s retail division. Consequently, the MNE will opt for the e-commerce entry model with “E only,” which possesses high initial market potential, followed by the model “E+FBP+SOP,” characterized by low initial market potential, and finally, the model “E+FBP,” which has medium initial market potential. The rationale is that when the service disparity between e-retailers and the retail segment of the MNE diminishes, it becomes challenging for the MNE’s retail division to secure a substantial competitive advantage. The MNE’s introduction of blockchain technology will lead to a more significant rise in consumers’ trust in e-commerce entry models with low initial market potential, particularly for e-retailer products. Thus, the MNE will lose part of their retail margins and incur blockchain technology costs. Moreover, while e-retailers will augment their orders from the manufacturing division of the MNE and wholesale profits will rise to a degree, the presence of tax disparity will exacerbate the tax liability of the MNE, and the reduction in tax planning advantage will diminish the increase in wholesale profits. Consequently, when the service disparity between e-retailers and the manufacturing division of the MNE is minimal, selecting an e-commerce entry strategy with moderate initial market potential can preserve retail profits while stabilizing wholesale profits.

### Policy recommendations

The robust growth of cross-border e-commerce has made selecting an e-commerce entry model a critical consideration for MNEs seeking to enter the online market while presenting new hurdles to their manufacturing divisions. Simultaneously, customer utility and preferences, tax disparities, and blockchain technology are vital in determining the e-commerce entry model for MNEs. Consequently, in light of the findings, this research proposes the subsequent recommendations:

(1) When estaablishing manufacturing and retail divisions, MNEs should consider the implications of tax disparities, refraining from seeking tax planning advantages that may result in situating the retail division in the jurisdiction with the lowest possible tax, which could lead to unnecessary losses due to the ALP regulation. Tax disparity is a paradoxical phenomenon. MNEs can exploit tax disparities to enhance tax planning by reducing the transfer price of their manufacturing division and channelling greater profits to their retail division in a low-tax area; however, the ALP regulation necessitates that MNEs concurrently decrease the wholesale price of their products to e-retailers, leading to diminished wholesale profits. Simultaneously, the E-retail industry is motivated to grow, hindering the MNEs’ manufacturing division from achieving greater anticipated profits from product sales. At the same time, the substantial tax burden would further diminish the MNEs’ retail earnings.
Furthermore, irrespective of the adoption of blockchain technology, MNEs must consider the implications of services rendered through the self-operated e-commerce entry model, the offerings from the MNE’s manufacturing division, the service sensitivity coefficient of consumers, and the influence of the MNE’s involvement in blockchain on consumer preferences regarding product value and service preferences. This understanding is essential for MNEs to modify their strategic decisions concerning e-commerce entry models adeptly.
(2) MNEs ought to select a monotypic e-commerce entry model, such as “E only” or “FBP only,” without the introduction of blockchain technology; conversely, they should adopt a composite e-commerce entry model, such as “E+FBP” or “E+FBP+SOP,” when blockchain technology is present. As a new digital financial innovation, Blockchain technology ensures quality assurance for e-retailers’ items through its robust product tracking capabilities, enhancing consumer confidence. The impact of heightened trust levels is contingent upon the initial market potential of the e-retailer division. When the initial market potential of the e-retailer sector is substantial, the implementation of blockchain technology by the MNE has a negligible impact on consumer trust; conversely, when the initial market potential is minimal, the introduction of blockchain technology by the MNE significantly enhances consumer trust. If the MNE has not implemented blockchain technology, selecting a monotypic e-commerce entrance model will provide more total profits; conversely, if the MNE has adopted blockchain technology, opting for a composite e-commerce entry model would generate higher total profits.
(3) For MNEs that have introduced blockchain technology, if they notice a gradual enhancement in the service level of self-operated e-commerce, they should select an e-commerce entry model based on the service gap between the e-commerce merchants and the MNE’s manufacturing division, considering options with high, low, and medium initial market potential. When the service gap is substantial, MNEs opt for the “E only” e-commerce entry model, using the enhanced market potential of e-commerce to get increased product orders while simultaneously mitigating the adverse impact on the manufacturing division resulting from the introduction of blockchain technology by the MNE. When the service gap is moderate, the “E+FBP+SOP” e-commerce entry model is used to address the diminished market potential of the e-commerce sector and to establish a definitive customer preference for the manufacturing division while using tax disparities for strategic planning. When the service gap is minimal, use the “E+FBP” e-commerce entry model to preserve retail margins and stable wholesale profitability.

## Robustness analysis

### Blockchain technology costs

**Fig 16:**
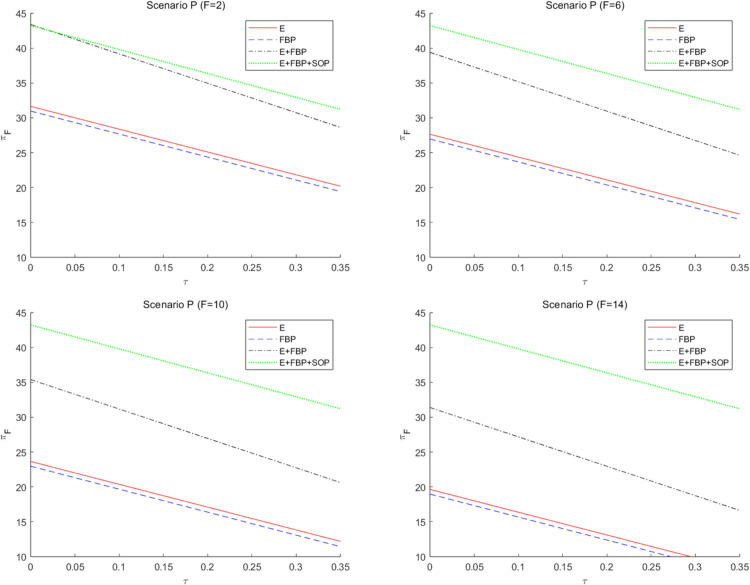
The impact of blockchain technology costs on the total profit of the MNE under variations in taxation.

In the baseline model, we set the introduction cost of blockchain technology as a fixed value *F* = 10. However, in practice, the implementation cost of blockchain technology varies significantly depending on product characteristics, supply chain complexity, technical solution choices, and regulatory requirements. To test the robustness of this paper’s core conclusions under varying blockchain technology costs and provide enterprises with more realistic decision-making references, this section treats blockchain technology cost *F* as a key variable. We explore how the heterogeneity of introduction costs affects the optimal strategy of the MNE that has already adopted blockchain technology. We set up four differentiated blockchain technology cost scenarios, with F∈{2,6,10,14}, to cover a range of application costs from low to high. Holding other benchmark parameters constant, we simulated how the total profit of the MNE fluctuates with tax differences when selecting different e-commerce entry models under these four cost levels. The results are shown in Fig 16.

Regarding the first conclusion, the four sets of simulation results provide highly consistent evidence. Across all scenarios, regardless of the e-commerce entry model adopted by the MNE, its total profits exhibit a significant monotonically decreasing trend as tax differentials increase. The magnitude of blockchain technology introduction costs does not alter the negative impact mechanism or direction of tax differentials on profits.

Furthermore, the second conclusion receives robust support from the four simulation sets, demonstrating that it is insensitive to the value of parameter F. Across all four simulations, profit curves for singular e-commerce entry models—such as “E only” or “FBP only”—consistently remain at low levels, with values closely aligned. This implies that neither single-type model can effectively enhance total corporate profits through blockchain technology, failing to gain a significant advantage. Conversely, the profit curve for the “E+FBP+SOP” model consistently occupies the highest position across all four scenarios. Its lead over other models expands linearly as *F* increases, while profits for the “E + FBP” or single-type models decline proportionally with rising blockchain implementation costs. This conclusively demonstrates that the higher the blockchain implementation cost, the more pronounced the comprehensive advantages of the composite e-commerce entry model become. Once the MNE decides to bear blockchain introduction costs—regardless of their level—shifting to a composite e-commerce entry model, particularly the “E+FBP+SOP” model with lower initial market potential, represents the optimal strategy for maximizing total profits.

In summary, variations in blockchain implementation costs primarily affect the absolute levels of profit curves but do not alter the relative ranking of different models or the core economic principles. Consequently, the core conclusions of this paper exhibit robust stability and are not contingent on specific parameter values.

### Consumer valuation distribution

The baseline model in this paper assumes that consumers’ valuation of a product, denoted as v, follows a uniform distribution over the interval (0, 1). This standard assumption ensures the model’s simplicity and analytical tractability. However, in reality, the distribution of consumers’ willingness to pay may be more concentrated around a mean, such as a bell-shaped distribution. To test whether the conclusions of this paper depend on the specific assumption of uniformity, we replace the distribution of consumer valuation v with a truncated normal distribution. Specifically, we define a distribution where consumer valuations follow a mean of $\bar{v}$, a standard deviation of σ, and are truncated on the interval (0,1). Its probability density function is given by Eq 33.


$$f(v)=\frac{\Phi \left(\frac{v-\bar{v}}{\sigma }\right)}{\sigma \left[\Phi \left(\frac{1-\bar{v}}{\sigma }\right)-\Phi \left(\frac{0-\bar{v}}{\sigma }\right)\right]},\;v\in (0,1)$$ (33)

Among these, ϕ(•) and Φ(•) represent the probability density function and cumulative distribution function of the standard normal distribution, respectively.

We set two representative parameter sets to capture distinct market structures. *TruncNormal*_*A*_ has a mean $\bar{v}$=0.5 and standard deviation σ=0.25, representing a symmetric market analogous to mass-market consumer goods or standard commodity markets. In this scenario, consumer valuations cluster around the midpoint. Compared to a uniform distribution, the proportion of consumers with extremely high or low willingness-to-pay decreases. *TruncNormal*_*B*_ has a mean of $\bar{v}$=0.65 and a standard deviation of σ=0.2, representing a high-end market. This can be compared to categories where consumers exhibit higher willingness-to-pay and the market is relatively premium. This scenario simulates a market with overall higher consumer willingness-to-pay and a more concentrated distribution.

**Fig 17:**
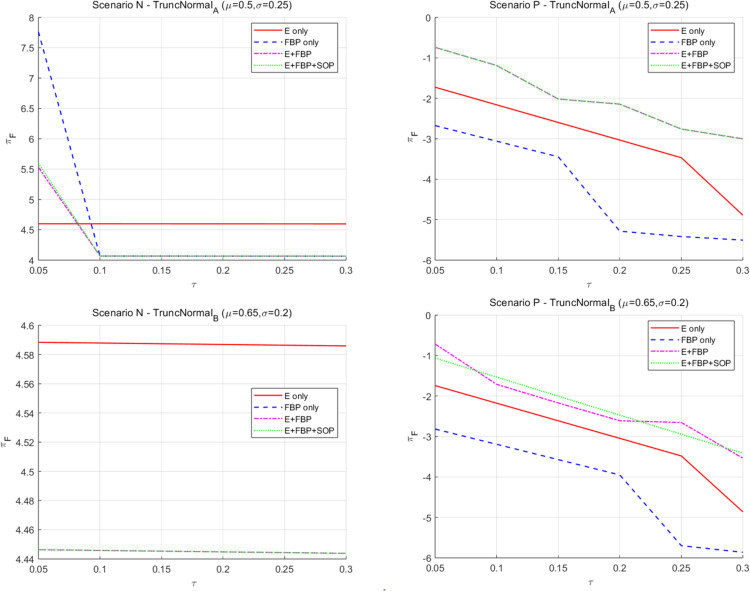
Under two truncated normal distributions, the trend of total profits for the MNE across four e-commerce entry models as tax differentials vary, with and without the introduction of blockchain technology.

Since closed-form solutions for equilibrium cannot be obtained after distribution changes, we employ a combined approach of Monte Carlo simulation and numerical optimization to solve the game equilibrium. Under the benchmark model parameter set, we fix all other parameters consistent with the numerical analysis section. For the two distribution scenarios above, we separately solve the equilibrium decisions for manufacturers and retailers and calculate the profits for each party. Through Monte Carlo simulation of 500,000 consumer counts, we conducted numerical case studies. Fig 17 respectively illustrate the trend of the MNE’s total profit across four e-commerce entry models, varying with tax differential, under two truncated normal distributions—both with and without blockchain technology introduced.

First, the numerical case study analysis provides strong support for the first conclusion. We observe that under both distributions, whether or not the MNE adopts blockchain technology, its total profits decrease as tax differentials narrow. This indicates that regardless of whether consumer valuations follow a uniform distribution or two truncated normal distributions, the trend of the MNE’s profits monotonically declining with tax differentials remains robust. Second, regarding the second conclusion, we still arrive at consistent results at the qualitative level. As shown in the figure, although the absolute value of the MNE’s total profit shifts with changes in distribution, the composite e-commerce entry model still demonstrates superior performance when the MNE has adopted blockchain technology. This further validates the robustness of this paper’s conclusions.

This analysis indicates that while alterations in consumer valuation distribution shapes quantitatively affect specific equilibrium strategy thresholds and profit levels, the core conclusions derived in this paper remain qualitatively robust. This demonstrates that the core findings are not special cases arising from the uniform distribution assumption, thereby enhancing the universality of our research conclusions.

## Future research

### Discussion and extension of nonlinear models

The core model of this paper follows the classical framework of vertical differentiation research in industrial organization theory, employing a linear additive form of the consumer utility function, as shown in Eq 1. The primary advantage of this standard assumption lies in its ability to derive closed-form solutions for complex Stackelberg games involving multiple participants. This enables a clear and definitive revelation of the intrinsic logical relationship between blockchain technology impacts and the MNE’s decisions on e-commerce entry models, facilitating rigorous comparative static analysis. Thus, the linear model established herein serves as a robust and necessary theoretical benchmark. However, the linear assumption implies constant marginal utility for consumers regarding both blockchain-enhanced value $\lambda \theta_{i}v$ and services $\mu \beta \;s_{i}$, potentially oversimplifying the complexity of real-world consumer behavior. To explore the robustness of the model’s conclusions and indicate future directions, we now discuss a more general nonlinear formulation, as shown in Eq 34.


$$U_{i}=\phi (\lambda \theta_{i}v)-P_{i}+\Psi (\mu \beta s_{i})$$ (34)

Among these, ϕ(x)=ln(1+x), $\Psi \left(x\right)=\sqrt{x}$, and satisfy ϕ′>0,$\phi^{\prime \prime }<0$, Ψ′>0, $\Psi^{\prime \prime }<0$.

Concave functions ∅(x) and Ψ(x) imply that blockchain technology relatively weakens its preference-enhancing effect for high-valuation consumers and its utility-enhancing effect for high-service-level merchants, altering the attractiveness of different market segments. Specifically, in the linear model, blockchain uniformly increases all consumers’ willingness to pay for e-retail channels by λ. Under nonlinear settings, its relative enhancement effect for mid-to-low valuation consumers may be more pronounced, aligning with our linear model findings. This divergence could influence market segmentation boundaries between e-retailers and the MNE’s retail divisions, thereby subtly altering pricing strategies and channel conflict intensity. The introduction of blockchain technology similarly influences consumer preferences for services. It is foreseeable that certain monotonic conclusions regarding λ and μ established under linear models may require additional conditions in nonlinear scenarios.

### Blockchain parameter endogenization

The benchmark model of this study treats the enhancement effects of blockchain on consumer valuation preferences λ and service perceptions μ, along with its implementation costs F, as exogenous parameters. However, in global digital trade practices, the MNE faces a highly heterogeneous global regulatory environment, where countries differ in their legal recognition, regulatory stances, and supportive policies toward blockchain. Therefore, future research could construct a two-stage dynamic game framework. This framework would translate the regulatory environment of target markets into a multidimensional composite variable R, representing “Regulatory Friendliness.” It would also transform the enhancement effects of blockchain on consumer valuation preferences λ and service perceptions μ, along with its implementation cost F, from exogenous settings into endogenous strategic choices made by the MNE in forward-looking decision-making. This approach would enable the study of corporate technology adoption strategies under regulatory uncertainty.

The first stage of the game involves endogenous investment decisions on technology. The “Regulatory Friendliness” variable R can be constructed by integrating policy indicators specific to the blockchain sector, such as the clarity of cross-border data flow regulations, the legal status of digital assets, and the degree of international mutual recognition of technical standards. In this phase, the MNE, acting as leaders, first makes strategic choices regarding blockchain technology solutions based on its assessment of the target market’s regulatory friendliness R, technological ecosystem, and consumer endowments. The core of its decision lies in selecting an optimal “technology efficiency-cost” combination, namely $\lambda^{*}$, $\mu^{*}$, F^*^. This can be achieved by defining a technology conversion cost function modulated by the regulatory environment, as shown in Eq 35.


F=C(λ,μ;R) (35)

Among these, $\frac{\partial C}{\partial \lambda }>0$,$\frac{\partial C}{\partial \mu }>0$, and C(•) is a convex function.

This function indicates that introducing blockchain technology to achieve higher consumer valuation preferences λ, and service-perceived efficiency gains μ requires paying higher marginal costs. A favorable regulatory environment, represented by a higher R, may flatten the cost function curve, enabling higher λ and μ to be obtained at the same cost. At this point, the decision-making problem for the MNE transforms into a constrained optimization problem. Within the set of technologically feasible combinations (λ, μ, F) | *F* = C(λ, μ, R), select the combination that maximizes the anticipated total profit across all phases. The second phase of the game involves decision-making on e-commerce entry models under given technical parameters. After the blockchain technology introduction strategy is selected—that is, after λ, μ, and *F* are determined—the game proceeds to the e-commerce entry model decision phase already solved in this paper. Given the endogenously selected blockchain parameters, the MNE interacts with its retail division and various e-retailers according to the Stackelberg game model established in Chapter 3 of this paper, and the optimal expressions are solved. The equilibrium profit function $\pi_{F}^{*}\left(\lambda ,\mu ,F\right)$ generated in this phase serves as the objective function for evaluating the relative merits of all potential technology combinations identified in the first stage. This modeling approach transforms “regulatory uncertainty” from a contextual description into an analyzable core variable. Its theoretical value lies in revealing the core mechanism of bidirectional shaping and co-evolution between “technological systems” and “institutional systems.” Through the endogenous R variable, the model depicts how technological innovation generates regulatory demands, while forward-looking institutional arrangements carve out “legitimacy space” and market pathways for technological innovation. This micro-level modeling helps reveal the macro-level trend of converging technical standards and regulatory standards within digital globalization.

### Limitations of blockchain symmetricity impact

This paper posits that blockchain technology enhances consumers’ perceived valuation of products and services across various e-retailers through unified coefficients λ and μ. This represents a necessary simplification for the baseline model. Its core purpose is to isolate the complex effects of blockchain technology’s potential heterogeneous implementation across channels or consumer trust heterogeneity. By doing so, we can focus on analyzing blockchain’s core mechanisms of “quality verification” and “service empowerment.” We investigate how the introduction of blockchain technology alters the overall competitiveness of online channels, thereby interacting with key factors like tax differentials and channel structure to influence the MNE’s strategic decisions regarding e-commerce entry models. This framework enables us to clearly reveal the core logic chain of “blockchain adoption → enhanced overall image of online channels → altered equilibrium in supply chain games,” laying a theoretical foundation for understanding more complex scenarios.

However, in reality, the credibility enhancement effect of blockchain may vary due to differences in platform governance models, inherent trust levels among various e-retailers, or consumers. For instance, consumers may place greater trust in blockchain traceability information provided by well-known platforms’ proprietary operations while maintaining reservations toward equivalent information from SOP merchants. Future research could relax this symmetry assumption by introducing heterogeneous blockchain impact coefficients, such as $\lambda_{E}$,$\lambda_{A}$,$\lambda_{B}$. This would explore how blockchain technology reshapes the relative competitive landscape among different types of e-retailers, thereby enhancing the contextual nuance and practical guidance of research conclusions.

### Real-world challenges and limitations in blockchain technology implementation

Although this paper focuses on the enabling role of blockchain technology in quality verification and trust building, we must acknowledge that it still faces numerous challenges in enterprise-level applications. First, technical limitations exist in scalability, throughput constraints, and energy consumption, particularly for public blockchains. Second, the “garbage in, garbage out” principle implies that the authenticity of on-chain data heavily depends on the mechanisms for initial information entry—a process itself requiring additional governance and audit costs. Furthermore, the success of cross-organizational blockchain implementation largely hinges on ecosystem coordination capabilities and incentive alignment design [24]. Sodhi and Tang (2019) [25] research has demonstrated that blockchain projects may face difficulties in advancement or fail to achieve expected outcomes when power imbalances exist among participants or when business objectives are misaligned. Therefore, beyond evaluating the explicit costs (F) discussed herein, enterprises must prudently assess implicit costs related to technological maturity, partnership dynamics, and process restructuring when making decisions.

### From vertical competition to ecosystem rivalry

This study constructs a vertical Stackelberg game model featuring the MNE as a leader and its retail division alongside various e-retailers as followers, focusing on vertical control and coordination issues. However, real-world cross-border e-commerce ecosystem competition is far more complex. Future research could build upon this model by incorporating additional horizontal and networked competitive dimensions, extending the model further upstream and downstream within the supply chain, and including a broader range of ecosystem participants to develop a more panoramic analytical framework. For instance, examining the impact of third-party service providers’ quality and cost, or the role of social media as a traffic driver for e-commerce products. Investigating how these ecosystem actors indirectly modulate the competitiveness and profit structure of the MNE’s online channels by influencing end consumers’ service experiences, trust valuations, and post-sale efficiency would better reveal the intricate collaborative mechanisms within digital supply chain ecosystems.

## Appendix

### Proof of A1

This section uses the “E only” model as an example to fully present the derivation process of the consumer demand function and the optimal solutions for Proposition 1. The consumer utility function is shown in Eq 1. Assuming consumer valuation v~U(0,1), the demand function can be obtained by comparing utilities. The indifference point *v*_1_ satisfies *U*_*R*_ = *U*_*E*_, i.e., $v-P_{R}+\beta s_{R}=\lambda \theta_{E}v-P_{E}+\mu \beta s_{E}$. Solving yields $v_{1}=\frac{P_{R}-P_{E}+\beta \left(\mu s_{E}-s_{R}\right)}{1-\lambda \theta_{E}}$. Additionally, consumers purchase only when the utility is non-negative. For the MNE’s retail division, consumers purchase its products when *U*_*R*_ ≥ 0, i.e., $v\geq P_{R}+\beta s_{R}$. For the self-operated e-commerce platform, the purchase condition is $v\geq \frac{P_{E}-\mu \beta s_{E}}{\lambda \theta_{E}}$. Assuming market coverage, the demand function is given by Eq 36.


$$\left\{ \begin{array}{c} D_{R}=1-v_{1}\\ {}[1ex]D_{E}=v_{1}-\frac{P_{E}-\mu \beta s_{E}}{\lambda \theta_{E}} \end{array}\right.$$ (36)

#### Retail-stage price competition.

Given wholesale price w, the MNE’s retail division and its self-operated e-commerce platform simultaneously determine retail prices *P*_*R*_ and *P*_*E*_ to maximize their respective profits. Take the first derivative of *P*_*R*_ and *P*_*E*_ separately and set them equal to zero, as shown in Eq 37.


$$\left\{ \begin{array}{c} \frac{\partial \pi_{R}}{\partial P_{R}}=D_{R}+(P_{R}-w)\frac{\partial D_{R}}{\partial P_{R}}=0\\ {}[3ex]\frac{\partial \pi_{E}}{\partial P_{E}}=D_{E}+(P_{E}-w)\frac{\partial D_{E}}{\partial P_{E}}=0 \end{array}\right.$$ (37)

Substituting into the demand function and solving the linear equations yields the reaction function for retail prices, as shown in Eq 38.


$$\left\{ \begin{array}{c} P_{R}=\frac{1}{2}\left(1+\beta s_{R}+w-\frac{\beta (\mu s_{E}-s_{R})}{1-\lambda \theta_{E}}\right)\\ {}[3ex]P_{E}=\frac{1}{2}\left(\lambda \theta_{E}+\mu \beta s_{E}+w-\frac{\lambda \theta_{E}\beta (\mu s_{E}-s_{R})}{1-\lambda \theta_{E}}\right) \end{array}\right.$$ (38)

#### Manufacturing sector wholesale price decision.

Substituting the demand function Eq 36 and the retail price reaction function Eq 38 into the MNE total profit function $\pi_{F}=w\left(D_{R}+D_{E}\right)\left(1-\tau \right)+\pi_{R}-F$ yields the single-variable function $\pi_{F}\left(w\right)$ with respect to *w*. Taking the first derivative with respect to *w* gives $\frac{d\pi_{F}}{dw}=0$. Solving this equation yields the optimal wholesale price w^*^, expressed as in Eq 4 in the main text. Substituting w^*^ back into the retail price reaction function yields the optimal retail prices $P_{R}^{*}$ and $P_{E}^{*}$, expressed as in Eq 4, Eq 5, and Eq 6 in the main text. Finally, substituting w^*^, $P_{R}^{*}$ and $P_{E}^{*}$ into the MNE total profit function yields the optimal total profit $\pi_{F}^{*}$.

### Proof of A2

This section uses the “E only” model as an example to rigorously prove that, within the parameter range satisfying the model’s fundamental assumptions, the Stackelberg game under this model possesses a unique subgame-refined Nash equilibrium interior point solution. Furthermore, this solution satisfies the second-order conditions for optimality. The proof consists of two parts concerning the concavity of Stage 2 retailer profits and the concavity of Stage 1 MNE profits.

#### Phase II: Concavity of retailer profit functions and nash equilibrium existence.

Given the wholesale price *w*, the MNE’s retail division engages in price competition with the self-operated e-commerce platform. Their profit functions are defined by Eq 3 $\pi_{R}=\left(P_{R}-w\right)D_{R}$ and $\pi_{E}=\left(P_{E}-w\right)D_{E}$. Here, demand functions *D*_*R*_ and *D*_*E*_ are defined by Eq 2. For simplicity, let constants $K=1-\lambda \theta_{E}$ and $M=\beta \left(\mu \;s_{E}-s_{R}\right)$. Given the assumptions $0<\theta_{E}<1$ and $1<\lambda <\frac{1}{\theta_{E}}$, we obtain K > 0.

The demand function can be rewritten as Eq 39.


$$\left\{ \begin{array}{c} D_{R}=1-\frac{P_{R}-P_{E}+M}{K}\\ {}[2ex]D_{E}=\frac{\lambda P_{R}\theta_{E}-P_{E}+\beta (\mu s_{E}-\lambda \theta_{E}s_{R})}{\lambda \theta_{E}K} \end{array}\right.$$ (39)

1. Concavity of the Profit Function with Respect to Its Own Price
  For the retail sector, we have Eq 40.

$$\left\{ \begin{array}{c} \frac{\partial \pi_{R}}{\partial P_{R}}=D_{R}+(P_{R}-w)\frac{\partial D_{R}}{\partial P_{R}}=D_{R}-\frac{P_{R}-w}{K}\\ {}[3ex]\frac{\partial^{2}\pi_{R}}{\partial P_{R}^{2}}=\frac{\partial D_{R}}{\partial P_{R}}\cdot \frac{1}{K}=-\frac{1}{K}-\frac{1}{K}=-\frac{2}{K}<0 \end{array}\right.$$ (40)

Since K > 0, the second derivative is always negative, hence $\pi_{R}$ is strictly concave with respect to *P*_*R*_.

(2) For the self-operated e-commerce segment, we have Eq 41.

$$\left\{ \begin{array}{c} \frac{\partial \pi_{E}}{\partial P_{E}}=D_{E}+(P_{E}-w)\frac{\partial D_{E}}{\partial P_{E}}=D_{E}-\frac{P_{E}-w}{\lambda \theta_{E}K}\\ {}[3ex]\frac{\partial^{2}\pi_{E}}{\partial P_{E}^{2}}=\frac{\partial D_{E}}{\partial P_{E}}\cdot \frac{1}{\lambda \theta_{E}K}=\frac{1}{\lambda \theta_{E}K}-\frac{1}{\lambda \theta_{E}K}=0 \end{array}\right.$$ (41)

Since $\lambda \theta_{E}>0$ and *K* > 0, the second derivative is always negative, so $\pi_{E}$ is strictly concave with respect to *P*_*E*_.

1. Negative Definiteness of Hessian Matrix and Uniqueness of Nash Equilibrium

The profit functions of the MNE’s retail divisions and their self-operated e-commerce platforms form a two-dimensional optimization system. Its Hessian matrix H is defined as Eq 42.


$$H=[\begin{array}{cc} \frac{\partial^{2}\pi_{R}}{\partial P_{R}^{2}} & \frac{\partial^{2}\pi_{R}}{\partial P_{E}\partial P_{R}}\\ {}[2ex]\frac{\partial^{2}\pi_{E}}{\partial P_{E}\partial P_{R}} & \frac{\partial^{2}\pi_{E}}{\partial P_{E}^{2}} \end{array}]$$ (42)

where the cross-partial derivatives are given by $\frac{\partial^{2}\pi_{R}}{\partial P_{R}\partial P_{E}}=\frac{\partial D_{R}}{\partial P_{E}}=\frac{1}{K}$, $\frac{\partial^{2}\pi_{E}}{\partial P_{E}\partial P_{R}}=\frac{\partial D_{E}}{\partial P_{R}}=\frac{1}{K}$. Therefore, the Hessian matrix H can be expressed as Eq 43.


$$H=[\begin{array}{cc} -\frac{2}{K} & \frac{1}{K}\\ {}[2ex]\frac{1}{K} & -\frac{2}{\lambda \theta_{E}K} \end{array}]$$ (43)

The principal minor of order one is $D_{1}=-\frac{2}{K}<0$. The second-order principal minor is $D_{2}=det\left(H\right)=\left(-\frac{2}{K}\right)\left(-\frac{2}{\lambda \theta_{E}K}\right)-{\left(\frac{1}{K}\right)}^{2}=\frac{4}{\lambda \theta_{E}K^{2}}-\frac{1}{K^{2}}=\frac{4-\lambda \theta_{E}}{\lambda \theta_{E}K^{2}}$. From the assumption $1<\lambda <\frac{1}{\theta_{E}}$, we obtain $0<\lambda \theta_{E}<1$. Therefore, $4-\lambda \theta_{E}>0$, implying *D*_2_ > 0.

The Hessian matrix H is negative definite if and only if all its sequential principal minors alternate in sign (negative for odd-order minors, positive for even-order minors). We have verified that *D*_1_  <  0,*D*_2_ > 0, thus H is negative definite. This guarantees that the second-stage game possesses a unique optimal reaction function. Furthermore, the Nash equilibrium $P_{R}^{*}$ and $P_{E}^{*}$ derived from its simultaneous equations is a unique and stable interior-point solution.

#### Phase I: Concavity of the profit function for the MNE.

Substitute the optimal response function obtained in the second stage from Eq 5 and Eq 6 in Proposition I—namely, $P_{R}^{*}(w)$ and $P_{E}^{*}(w)$—into the MNE’s total profit function $\pi_{F}^{*}=w^{*}\left(\frac{P_{E}^{*}-\beta s_{E}\mu }{\theta_{E}\lambda }-1\right)\left(\tau -1\right)-\left(\frac{P_{E}^{*}-P_{R}^{*}+\beta \left(s_{R}-s_{E}\mu \right)}{\theta_{E}\lambda -1}-1\right)\left(P_{R}^{*}-w^{*}\right)-F$. Since both $P_{R}^{*}\left(w\right)$ and $P_{E}^{*}\left(w\right)$ are linear functions of *w*, $\pi_{F}^{*}(w)$ is actually a quadratic function of w. Therefore, its concavity depends on the sign of the quadratic coefficient. Substituting the linear expressions $P_{R}^{*}\left(w\right)=a_{1}w+b_{1}$, and $P_{E}^{*}\left(w\right)=a_{2}w+b_{2}$ into $\pi_{F}^{*}(w)$ and simplifying yields the general form $\pi_{F}^{*}\left(w\right)=Aw^{2}+Bw+C$. Here, the coefficients *a*_1_,*a*_2_,*A*, and *B* are functions of the model parameters. Direct calculation yields the quadratic coefficient $A=\frac{\left(\tau -1\right)\left(4-3\lambda \theta_{E}\right)}{\lambda \theta_{E}{\left(4-\lambda \theta_{E}\right)}^{2}}-\frac{2\left(2-\lambda \theta_{E}\right)}{\left(1-\lambda \theta_{E}\right){\left(4-\lambda \theta_{E}\right)}^{2}}$. Under the model assumptions $0<\theta_{E}<0$, $1<\lambda \theta_{E}<1$, 0<τ<1, it can be proven that the above expression A < 0. Therefore, $\pi_{F}^{*}(w)$ is strictly concave with respect to w, and its first-order condition $\frac{d\pi_{F}^{*}}{dw}=0$ uniquely determines the maximum point, which is the optimal wholesale price w^*^ in Proposition 1.
